# A systematic review of reviews identifying UK validated dietary assessment tools for inclusion on an interactive guided website for researchers: www.nutritools.org

**DOI:** 10.1080/10408398.2019.1566207

**Published:** 2019-03-18

**Authors:** Jozef Hooson (Jzh), Jayne Hutchinson (Jyh), Marisol Warthon-Medina, Neil Hancock, Katharine Greathead, Bethany Knowles, Elisa Vargas-Garcia, Lauren E. Gibson, Linda A. Bush, Barrie Margetts, Sian Robinson, Andy Ness, Nisreen A. Alwan, Petra A. Wark, Mark Roe, Paul Finglas, Toni Steer, Polly Page, Laura Johnson, Katharine Roberts, Birdem Amoutzopoulos, Victoria J. Burley, Darren C. Greenwood, Janet E. Cade

**Affiliations:** a Nutritional Epidemiology Group, School of Food Science and Nutrition, University of Leeds, Leeds, UK;; b Food Databanks National Capability, Quadram Institute Bioscience, Norwich, UK;; c Faculty of Medicine, University of Southampton, Southampton, UK;; d MRC Lifecourse Epidemiology Unit, University of Southampton, Southampton, UK;; e NIHR Southampton Biomedical Research Centre, University of Southampton & University Hospital Southampton NHS Foundation Trust, Southampton, UK;; f NIHR Biomedical Research Unit in Nutrition, Diet and Lifestyle, University Hospitals Bristol NHS Foundation Trust and the University of Bristol, Bristol, UK;; g Academic Unit of Primary Care and Population Sciences, Faculty of Medicine, University of Southampton, Southampton General Hospital, Southampton, UK;; h Centre for Innovative Research Across the Life Course (CIRAL), Faculty of Health and Life Sciences, Coventry University, Coventry, UK;; i Global eHealth Unit, Department of Primary Care and Public Health, Imperial College London, London, UK;; j EuroFIR AISBL, Brussels, Belgium;; k MRC Elsie Widdowson Laboratory, Cambridge, UK;; l Centre for Exercise, Nutrition and Health Sciences, School for Policy Studies, University of Bristol, Bristol, UK;; m Public Health Section, School of Health and Related Research (ScHARR), University of Sheffield, Sheffield, UK;; n Public Health England, London, UK;; o Faculty of Medicine and Health Division of Biostatistics, University of Leeds, Leeds, UK

**Keywords:** Validation studies, Diet records, Systematic Review, Study Characteristics, Dietary Assessment, Limits of Agreement

## Abstract

**Background:** Health researchers may struggle to choose suitable validated dietary assessment tools (DATs) for their target population. The aim of this review was to identify and collate information on validated UK DATs and validation studies for inclusion on a website to support researchers to choose appropriate DATs.

**Design:** A systematic review of reviews of DATs was undertaken. DATs validated in UK populations were extracted from the studies identified. A searchable website was designed to display these data. Additionally, mean differences and limits of agreement between test and comparison methods were summarized by a method, weighting by sample size.

**Results:** Over 900 validation results covering 5 life stages, 18 nutrients, 6 dietary assessment methods, and 9 validation method types were extracted from 63 validated DATs which were identified from 68 reviews. These were incorporated into www.nutritools.org. Limits of agreement were determined for about half of validations. Thirty four DATs were FFQs. Only 17 DATs were validated against biomarkers, and only 19 DATs were validated in infant/children/adolescents.

**Conclusions**: The interactive www.nutritools.org website holds extensive validation data identified from this review and can be used to guide researchers to critically compare and choose a suitable DAT for their research question, leading to improvement of nutritional epidemiology research.

## Introduction

Diets high in energy dense and nutrient-poor foods have been linked to an increased risk of chronic diseases such as obesity, cardiovascular disease, and particular cancers (Rollo et al. 2016). Measuring dietary intake accurately is, therefore, essential in establishing relationships between food consumption patterns and non-communicable diseases (Serra-Majem et al. 2009); or when evaluating the effectiveness of public health policies and interventions (Mouratidou et al. 2012; Øverby, Serra-Majem, and Andersen 2009). Accurate measurement of dietary intake, both at an individual and population level, is challenging due to measurement difficulties, low participation rates, and degree of compliance, with no single method being identified as the best approach for population studies (Shim, Oh, and Kim 2014).

Dietary measurement has relied on self-reported dietary assessment tools (DATs) such as food frequency questionnaires (FFQs), 24-hour recalls, and weighed/estimated food diaries (WFD, EFD) (Johnson 2002; Long et al. 2010). However, these methods are prone to selective underreporting, misreporting, are expensive, and may have low compliance (Shim, Oh, and Kim 2014; Bingham and Day 1997). Advancements in computer technology have helped address some of these issues (Cade 2017; Timon et al. 2016). However, it has been recognized that there is no universal DAT which is suitable for all dietary assessment research. A description of the main DATs used to assess dietary intake is shown in Supplementary Material, Table 1.

**Table 1.** Inclusion and exclusion criteria applied to the reviews and DATs.

Reviews		DATs	
Inclusion criteria	Exclusion criteria	Inclusion criteria	Exclusion criteria
Reviews that validated a DAT against a biomarker or another self-reported tool against energy, macro or micro nutrients or food groups Reviews published since 1st January 2000	Reviews that exclusively evaluated tools assessing inadequacy of diets in terms of malnutrition Commentaries, editorials or other opinion articles	Tools validated in a UK populationBe able to measure dietary intake Validation results can be entered on the nutritools website	DATs measuring eating disorders, food preferences, feeding practices or inadequacy of diets Lifestyle based tools (e.g. diet plus physical activity) DATS measuring the purchasing of foods / drinks Tools that assessed specific dietary interventions (e.g. Atkins, Mediterranean diet) Non-UK tools

A number of key factors should be considered when selecting the most suitable DAT, including the dietary component of interest, the characteristics of the population, the time frame required, the type and accuracy of data required, the food composition table used, and the resources available (Cade 2017). The tool should also be validated for the foods or nutrients of interest and in the population being measured. However, validation information may not be readily available to researchers and not all DATs are easily accessible for use.

The aim of this review was to identify and collate characteristics of DATs which have been validated in the UK population and to include this information together with characteristics of their validation studies and the validation results on the DIET@NET partnership project’s www.nutritools.org website. The aim of the website is to help researchers and health professionals critically compare and select the most suitable validated DATs for their research question which ultimately may lead to improvements in nutritional epidemiology research. An additional aim was to tabulate the validation results in this article to explore whether they varied by DAT type and reference method type.

## Methodology

A systematic review of reviews of DATs was undertaken to identify validated DATs. Literature reviews as well as systematic reviews were examined, as it was acknowledged that not all validated DATs would be identified through systematic reviews only. From the identified reviews, details of the associated development and validation papers for the UK-specific tools were extracted. An unpublished protocol was designed and agreed upon by members of the DIET@NET project.

### Search strategy

To identify reviews of validated DATs, the following bibliographic databases were searched: Cochrane Database of Systematic Reviews (CDSR); Database of Abstracts of Reviews of Effectiveness (DARE); National Health Service Economic Evaluation Database (NHS EED); Health Technology Assessment Database (HTA); Web of Science Core Collection; Ovid MEDLINE; In-Process; EMBASE; Scopus; CAB abstracts; and Open Grey. The search was initially conducted in May/June 2015, then updated in October 2016, and was restricted to reviews published between January 2000 and October 2016. No restriction was placed on when the tool was developed or validated. Reference lists of the selected reviews and relevant published conference proceedings were also searched. The search-strategy for MEDLINE is shown in Appendix 1. The search-strategy was adapted for other databases when Medical Subject Headings terms were unavailable. Citations were cataloged and managed within Endnote (X7).

### Selection of reviews

Two reviewers (JZH; KG) were independently involved in two rounds of screening to identify reviews that met the eligibility criteria. The first round of screening involved reviewing each article based on their title and abstract. Full copies of potential articles from the previous round were then downloaded for examination by both reviewers independently, to determine eligibility based on the inclusion and exclusion criteria. Any discrepancies between reviewers were reassessed and resolved by further discussion and advice from members of the Diet@Net project board.

### Tool identification from reviews

Papers relating to the original DAT development and/or validations identified in the reviews were downloaded and screened to determine eligibility for data extraction (BK). To be eligible for this stage of the review, the tools had to satisfy the inclusion criteria. The inclusion and exclusion criteria applied for both reviews and DATs are noted in Table 1. No date restriction was imposed on the actual tools or their developmental/validation papers. Online searches were carried out for each tool identified for further development or validation papers to ensure all relevant data were collected.

### Cross checking with other sources

It was acknowledged that not all UK validated DATs would be captured by our search strategy, as not all tools may have been included in a review published within the search years (2000–2016). This would particularly disadvantage in using more recent tools. Therefore, one reviewer (BK) cross checked against DAT registries which were The National Collaborative on Childhood Obesity Research (NCCOR) (https://tools.nccor.org/measures) and the National Cancer Institute (NCI): Dietary Assessment Primer (Dietary Assessment Calibration/Validation Register: “Find a Study”) (https://epi.grants.cancer.gov/cgi-bin/dacv/index.pl?page=stu dy_search). The Medical Research Council (MRC) website was checked for funded research on diet identifying particular DATs used, along with analyzing DATs from MRC-funded cohort studies.

### Data extraction from the developmental and validation papers and incorporation into website

Two researchers (JH; BK) extracted and collated data from the development and validation papers of the DATs in an Access database, and 10% was checked by a third investigator (KG). These data included characteristics of the DATs including lifestage of tool focus; how the tool was administered (by self, proxy or interview) and nutrient database used. Data on the DAT validation studies were also extracted, including the reference method used (e.g. 24 h recall, weighed food diary, biomarkers, and doubly labeled water) and time span of assessment. Results for validation of energy and 16 nutrients (total fat, saturated fat, monounsaturated fat, polyunsaturated fat, carbohydrate, protein, sugar, fiber (NSP), sodium, calcium, iron, zinc, retinol, folate, vitamin C, vitamin B12) plus fruit, and vegetables were extracted. The validation results comparing intakes estimated by the DAT and a reference method for the following statistical methods were extracted where available: mean difference and standard deviation, correlation coefficient, Cohen’s Kappa coefficient, percentage agreement, and Bland-Altman lower and upper limits of agreement. These data was then incorporated into the website www.nutritools.org. This website was designed and created by Xlab (www.x-labsystems.co.uk) based in Leeds, in collaboration with the Diet@Net team.

### Statistical analysis

Data were analyzed using Stata version 14 exploring the validation results by DAT and reference method type for energy and selected micro- and macronutrients to determine whether the validation results varied greatly by type of DAT or by the reference method, and to show the number of validations by lifestage and nutrient. For this the weighted mean of the differences in intakes (WMD) for each type was calculated, with larger samples having more influence on these summary results.

First the difference in the estimated nutrient intakes from each validation study was determined as the reference method value subtracted from the test DAT. Then, the number of individuals taking part in the validation studies was used to produce a weighted mean of these differences by tool and reference method type. Additionally, for each combination of reference method and tool, the range of the lower and upper Bland Altman limits of agreement (LOA) (Bland and Altman 1986) reported or calculated using the mean difference (MD) and standard deviations from the validation papers, was determined. We summarized these by three types of tools: food diary; 24 h dietary recall; FFQ/Food checklist, as these were the most common DAT types used. Diet histories were not included as there were only a small number of these and they are not commonly used in the UK. These were cross tabulated with four groups of reference measures: recovery biomarkers; food diary; 24-hour recall; FFQ. The results are displayed by two main lifestages: (i) infants, children and adolescents and (ii) adults and elderly.

## Results

**Figure 1.**
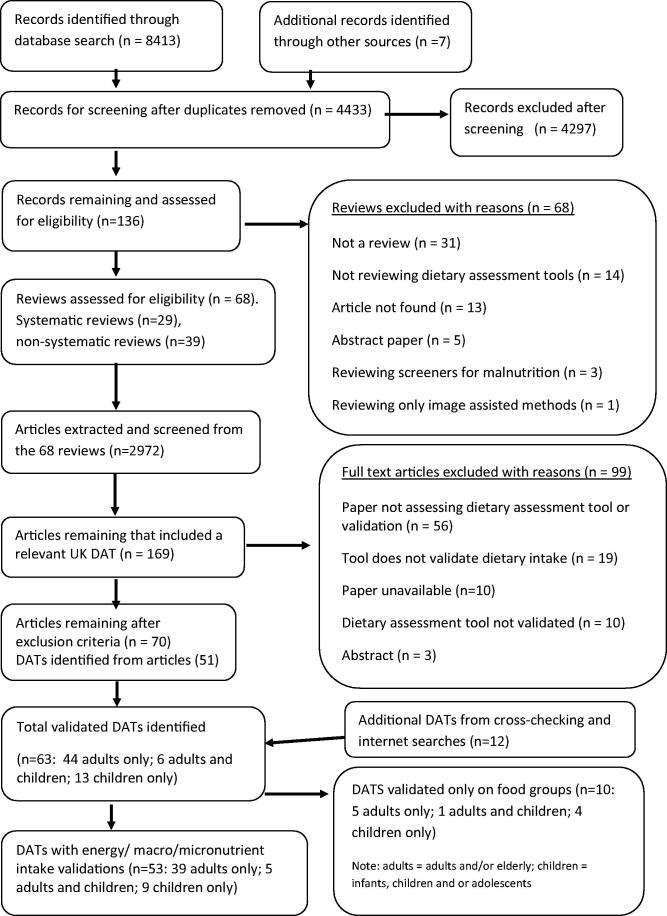
PRISMA flow chart indicating number of articles included at each phase.

A total of 8413 review articles were identified from the database searches (see Fig. 1). A further seven reviews were identified through reference tracking and Internet searches. After removing duplications, 4433 articles remained, with 4297 excluded after screening of the title and abstract. After screening the full texts of the 136 articles, 68 reviews remained; of which 29 (43%) were systematic and 39 (57%) were nonsystematic literature reviews. No review only reported tools that had been validated in a UK population. The main objective of the reviews varied, with some identifying tools validated for a specific population or lifestage, and others focusing on nutrient/food type. The characteristics of the reviews are shown in the Supplementary Material, Table 2.

**Table 2.** Number and description of dietary assessment tools for each life stage.

Validation life stage and number of tools	Description
Infants (≤3 yrs old) (*n* = 4)	2 FFQ and 2 food diaries
Children (3–11 yrs old) (*n* = 12)	5 recalls, 3 diaries, 2 checklists, 1 FFQ and 1 diet history
Adolescents (12–18 yrs old) (*n* = 10)	4 recalls, 2 food diaries, 2 food checklists, 1 FFQ and 1 diet history
*Pregnant women (*n* = 3)	All FFQ’s
Adults (age 19–64) (*n* = 47)	30 FFQ’s, 8 24-hour recalls, 6 food diaries, 2 food checklists and 1 diet history
Elderly (>65) (*n* = 19)	9 FFQ’s, 4 food diaries, 2 food checklists, 3 recalls and 1 diet history

*Also included in the adult cohort numbers.

Only 2 of the validation studies exclusively included participants >65.

Only 5 of the tools validated in children covered the full age range of 3–11 years old.

2 of the infant validated tools measured dietary intake for a specific infant age = 6 months and 12 months.

From the reviews, 2972 articles were extracted and screened. Only 169 (6%) of 2972 articles included a UK DAT that measured some aspect of diet, and 99 (59%) of these were excluded after full text screening (see Fig. 1 for reasons). From these 70 remaining articles, 51 different UK validated DATs were identified, with the review by Cade et al. (2004), providing the most with 24 (46%) validated DATs. Cross checking against DAT registries identified seven additional DATs with a further five identified from Internet searching and reference checking making a total of 63 DATs.

### Characteristics of the 63 DATs

Of the 63 DATs, 39 had macro- and micronutrient intakes validated in adult and/or elderly populations with a further five validated on all ages, and 19 DATs validated on infants/children and/or adolescents. Ten DATs focused only on food group intakes (5 adults/elderly only; 1 all ages; 4 infants/children and/or adolescents only). The majority of DATs validated on adults were FFQ, whereas those validated on children and adolescents were food checklists, diaries, or 24-hour recalls. The total number and description of the DATs for each separate life stage are shown in Table 2. Twelve (19%) of the 63 DATs were a modified version of a previously developed tool (Ashfield-Watt et al. 2007; Broadfield et al. 2003; Bingham et al. 1994; Bodner et al. 1998; Bolton-Smith et al. 1991; Brunner et al. 2001; Heath et al. 2005; Hillier et al. 2012; Johnson, Driscoll, and Goran 1996; Mouratidou, Ford, and Fraser 2006; Mckeown et al. 2001; Hooper et al. 2010), while the year the 63 DATs were developed ranged from 1981 to 2016.

The DAT characteristics are displayed in Table 3 along with their validation study characteristics; this information can also be found on the interactive website www.nutritools.org. The length of the 34 FFQs ranged from 8 to 630 food items/questions, with 13 (38%) of these classified as short FFQs consisting of ≤50 food questions/items and 10 (29%) classified as long FFQs consisting of >100 food questions/items. Of the 63 DATs, 16 (25%) were web-based tools by life stage and nutrient. Four tools focused on infants and toddlers (Lanigan et al. 2001; Marriott et al. 2009; Marriott et al. 2008; Davies et al. 1994). Twelve tools focused on children and 10 tools on adolescents. Forty-seven tools were developed to measure adult diet, and 19 were suitable for measuring diet in the elderly. The time frame covered by the DATs varied. Food diaries ranged from measuring intake over one day to repeated measures over one year. Most 24-hour recalls measured the previous 24-hours; however, some measured intakes over two consecutive or several days (e.g. Johansson 2008; Hillier et al. 2012; Johnson, Driscoll, and Goran 1996). FFQs ranged from the previous day to usual intake over the previous year with 11 (32%) measuring long-term intake (>6 months) and six (16%) measuring short-term intake (one day) (Ashfield-Watt et al. 2007; Bingham et al. 1994; Bingham and Day 1997; Broadfield et al. 2003; Brunner et al. 2001; Cleghorn et al. 2016). The food database underpinning the DATs was primarily a version of the McCance and Widdowson’s the Composition of Foods (MCW) food tables or a database based upon MCW. Of the DATs, 10 (16%) did not report the food database used; seven (70%) of these were FFQs.

**Table 3.** General characteristics of the 63 UK dietary assessment tools and their validation studies.

Dietary assessment tool validation studies									
First author (year)	Administration method / length of questionnaire	Nutrient database	First author and year	Food & nutrients (number of nutrients validated)	Life stage, age (mean /range) and *sample size (M/F)*	Reference method	Time span		Statistical method used
							DAT	Reference method	
Weighed Food Diary									
Bingham et al. (1994)	Self	MCW4	Bingham et al. (1997)	Urinary nitrogen Micronutrients (2)	Adults (50–65 yr) *156 (0/56)*	Biomarkers	16d	8d over 12 months	Individual Means; Correlation Coefficient (S); Cross Classification
Davies et al. (1994)	By-Proxy	MCW4	Davies et al. (1994)	Energy	Children & Infants (1.5 – 4.5 yr) *81 (42/39)*	DLW	4d consecutive	10d	Mean Difference; Correlation Coefficient; Limits of Agreement
Livingstone et al. (1992)	Self; By-Proxy	MCW4 Inc. supplementary food composition data	Livingstone (1992)	Energy	Children & Adolescents (7–18 yr) *58 (29/29)*	DLW	7d consecutive	10 – 14d	Mean Difference(%); Limits of Agreement
Estimated Food Diary									
Bingham et al. (1994)	Self	MCW4	Bingham et al. (1994)	Energy; Macronutrients (7); Micronutrients (6)	Adults (50–65 yr) *81 (0/81)*	Weighed Food Diary	7d	4 x 4d over 12 months	Individual Means; Correlation Coefficient (S); Cross Classification
			Bingham et al. (1997)	Urinary nitrogen Micronutrients (3)	Adults (50–65 yr) *80 (0/80*)	Biomarkers	7d	8d over 12 months	Correlation Coefficient (P)
			Johansson (2008)	Energy; Macronutrients (6); Micronutrients (6); Food Groups	Elderly (65–88 yr) *80 (80/0)*	Weighed Food Diary	7d	4 x 4d over 12 months	Individual Means
Carter et al. (2013) (mymealmate)	**Self	The Weight Loss Resources	Carter et al. (2013)	Energy; Macronutrients (3)	Adults (mean 35yr) *50 (14/36*)	24-Hour Recall	7d consecutive	2d	Mean Difference; Correlation Coefficient (P); Limits of Agreement
McKeown et al. (2001)	Self	DINER	McKeown et al. (2001)	Urinary nitrogen Micronutrients (3)	Adults & Elderly (45–74 yr) *146 (58/88)*	Biomarkers	7d	3d	Individual Means; Correlation Coefficient (P & S); Cross Classification
			Day	Micronutrient (2)	Adults (45–74yr) 123	Biomarkers	7d	6d over 12 months	Individual Means; Correlation Coefficient
Lanigan et al. (2001)	By-Proxy	COMP-EAT v.5	Lanigan et al. (2001)	Energy; Macronutrient (3)	Infants (6–24 months) *DLW = 21* *Weighed Food Diary =72*	DLW & Weighed Food Diary	5d	7d (DLW) & 5d (Food Diary)	Mean Difference (%); Limits of Agreement
Timon et al. (2015) (NANA method)	**Self	WinDiets	Timon et al. (2015)	Energy, Macronutrients (5); Micronutrients (10); Food Group	Elderly (65–89 yr) *94 (34/60)*	Estimated Food Diary & Biomarkers	4d	4d (Food Diary) & 1d (Biomarkers)	Mean Difference; Correlation Coefficient (P & S); Limits of Agreement
Semi-Weighed Food Diary									
Holmes, Dick, and Nelson (2008)	Self; By-Proxy; Interview	MCW5	Holmes, Dick, and Nelson (2008)	Energy; Macronutrients (4); Micronutrients (6); Food Group	Children, Adolescents, Adults, Elderly (2-90 yr) *44, 30, 111, 34* Low SES	Weighed Food Diary	4d	4d	Mean Difference.
24-hour recall									
*Bingham et al. (1994)	Self	MCW4	Bingham et al. (1994) (Structured & Unstructured)	Energy, Macronutrients (7); Micronutrients (6)	Adults (50–65 yr) *160 (0/160)*	Weighed Food Diary	1d	4 x 4d over 12 months	Individual Means; Correlation Coefficient (S); Cross Classification
			Bingham et al. (1997) (Structured & Unstructured)	Urinary nitrogen Micronutrients (3)	Adults (50–65 yr) *156 (0/156)*	Biomarkers	1d	8d over 12 months	Correlation Coefficient (P & S)
			Johansson (2008) (Unstructured)	Energy; Macronutrients (6); Micronutrients (6); Food Groups	Elderly (65–88 yr) *80 (80/0)*	Weighed Food Diary	7d	4 x 4d over 12 months	Individual Means
Carter et al. (2015) (myfood24)	**Self; Interview	MCW7	Albar et al. (2016)	Energy; Macronutrients (6); Micronutrients (1); Food Groups	Adolescents *75 (47/38)*	Multiple-Pass 24-Hour Recall	2d (non-consecutive)	2d (non-consecutive)	Mean Difference; Correlation Coefficient (ICC); Class Classification Limits of Agreement; Weighted Cohen’s kappa
*Comrie, Masson, and McNeill (2009) (FoRC)	**Self	MCW6	Comrie, Masson, and McNeill (2009)	Energy; Macronutrients (2); Food Groups	Adults (18–49 yr) *53 (12/41)*	Estimated Food Diary	4d	4d	Mean Difference; Correlation Coefficient (S); Limits of Agreement
Edmunds et al. (2002) (DILQ)	Self	Not Reported	Edmunds et al. (2002)	Food Groups	Children (7–9 yr) *204*	Direct Observation	1d	1d	Individual Means (count); Cross Classification (% matched); Cohen’s kappa
*Foster et al. (2014) [(INTAKE24)	**Self	MCW	Bradley et al. (2016)	Energy; Macronutrients (6); Micronutrients (3); Food Groups	Adolescents & Adults (11–24 yr) *168 (74/94)*	24-Hour Recall	4d (Results reported data on participants completing any number of days)	4d (Results reported data on participants completing any number of days)	Mean ratios; Limits of Agreement
Hillier et al. (2012) (SNAPA)	**Self	MCW6	Hillier et al. (2012)	Food Groups	Adults (mean 34) *44 (16/28)*	Direct Observation	5d	4d	Mean Difference; Cross Classification
*Holmes, Dick, and Nelson (2008)	By-Proxy; Interview	MCW5	Holmes, Dick, and Nelson (2008)	Energy; Macronutrients (4); Micronutrients (6); Food Group	Children, Adolescents, Adults, Elderly (2–90 yr) *76, 48, 206, 54* Low SES	Weighed Food Diary	4d	4d	Mean Difference.
*Johnson, Driscoll, and Goran (1996)	Interview	Food Intake Analysis	Reilly et al. (2001)	Energy	Children (3–4 yr) *41 (23/18)*	DLW	3d	7d	Mean Difference; Limits of Agreement
			Montgomery et al. (2005)	Energy	Children (4.5–7 yr) *63 (32/31)*	DLW	3d (Inc. 1 weekend d)	2d	Mean Difference (bias); Limits of Agreement
Little et al. (1999)	Interview	Not Reported	Little et al. (1999)	Macronutrients (1); Micronutrients (1); Food Groups	Adults & Elderly (18–80 yr) *111 (53/58)*	Weighed Food Diary	1d	7d	Median Difference (%) Correlation Coefficient (S)
Liu et al. (2011) (Oxford WebQ)	**Self	MCW5	Liu et al. (2011)	Energy; Macronutrients (9); Micronutrients (10); Food Group	Adults (19–82 yr) *116 (32/84)*	Multiple-Pass 24-Hour Recall	1d	1d	Mean Difference (%); Correlation Coefficient (S); Cross Classification
Moore et al. (2008) (SNAP)	**Self	Not Reported	Moore et al. (2008)	Food Groups	Children & Adolescents (7–15 yr) *121 (49/72)*	Multiple Pass 24-Hour Recall	1d	1d	Individual Means (Count); Cross Classification
Moore et al. (2007) (Dietary Recall Questionnaire)	**Self	Not Reported	Moore et al. (2007)	Food Groups	Children (9–11 yr) *374 (157/ 215)* Low SES	Multiple Pass 24-Hour Recall	1d & an extra morning	1d & an extra morning	Correlation Coefficient (S); Cross Classification; Cohen’s kappa
48-Hour Recall									
McNaughton et al. (2005)	Interview	MCW	McNaughton et al. (2005)	Energy, Macronutrients (4); Micronutrients (9) Food Group	Adults (43 yr) *2265 (1116/ 1149)*	Estimated Food Diary	2d	5d	Mean Difference; Correlation Coefficient (S)
Food Frequency Questionnaire									
Ashfield-Watt et al. (2007) (FACET)	Self ≤50 food items / questions	N/A	Ashfield-Watt et al. (2007)	Food Groups	Adults (age not reported) *269* Low SES	Estimated Food Diary	1d	1d	Individual Means; Correlation Coefficient; Cross Classification
Bingham et al. (1994) (Cambridge FFQ)	Self ≥100 food items / questions	MCW4	Bingham et al. (1994)	Energy; Macronutrients (7); Micronutrients (6) Food Groups	Adults (50–65 yr) *160 (0/160)*	Weighed Food Diary	1d	4 x 4d over 12 months	Individual Means; Correlation Coefficient (S); Cross Classification
Bingham et al. (1994) (Oxford FFQ)	Self ≥100 food items / questions	MCW4	Bingham et al. (1994)	Energy; Macronutrients (7); Micronutrients (6)	Adults (50–65 yr) *160 (0/160)*	Weighed food diary	1d	4 x 4d over 12 months	Individual Means; Correlation Coefficient (S); Cross Classification
			Bingham et al. (1997)	Micronutrients (3)	Adults (50–65 yr) *160 (0/160)*	Biomarkers	1d	8d over 12 months	Correlation Coefficient (P & S)
			Johansson (2008)	Energy; Macronutrients (6); Micronutrients (6); Food Groups	Elderly (65–88 yr) *80 (80/0)*	Weighed food diary	1d	4 x 4d over 12 months	Individual Means
			Samaras et al. (1998)	Energy; Macronutrients (4);	Adults (mean 58 yr) *162 (0/162)*	Estimated food diary	1d	7d	Individual Means; Correlation Coefficient
			Verkasalo et al. (2001)	Food Groups	Adults (20–39 yr) *80 (0/80)*	Biomarkers	1d	1d	Correlation Coefficient (S)
			Little et al. (1999)	Macronutrients (1); Micronutrients (1); Food Groups	Adults & Elderly (18–80 yr) *111 (53/58)*	Weighed Food Diary	1d	7d	Median Difference (%) Correlation Coefficient (S);
Broadfield et al. (2003) (DIETQ)	Self ≥100 food items / questions	DIETQ	Broadfield et al. (2003)	Macronutrients (5) Food groups	Adults (mean 42 yr) *31 (15/16)*	Weighed Food Diary	1d	7d	Mean Difference; Correlation Coefficient (P + S); Limits of Agreement
Brunner et al. (2001)	Self ≥100 food items / questions	MCW4 & MCW5	Brunner et al. (2001)	Energy Macronutrients (9); Micronutrients (8) Food Group	Adults (39–61yr) 860 *(457/403)*	Estimated Food Diary	1d	7d	Individual Means; Correlation Coefficient (S); Cross Classification
Cleghorn et al. (2016)	Self ≤50 food items / questions	DANTE	Cleghorn et al. (2016)	Macronutrients (1); Food Groups	Adults FFQ-*705 (314/ 391*); 24 hr Recall - *47 (25/22)*	FFQ & 24hr Recall	1d	1d	Mean Difference; Correlation Coefficient (S); Cohen’s kappa
Dunn et al. (2011) [30]	Self ≤50 food items / questions	Not reported	Dunn et al. (2011)	Macronutrients (2) Food Groups	Adults (18–50 yr) *66 (17/49)*	Weighed Food Diary	7d	7d	Mean Difference; Cross Classification; Limits of Agreement
Forster et al. (2014) (Food4Me)	Self ≥100 food items / questions	National Adult Nutrition Survey (NANS)	Forster et al. (2014)	Energy; Macronutrient (7); Micronutrients (14); Food Groups	Adults (30 yr) *113 (46/67)*	FFQ	1d	1d	Mean Difference; Correlation Coefficient (S); Class Classification; Limits of Agreement
			Fallaize et al. (2014)	Energy; Macronutrients (7); Micronutrients (13); Food Groups	Adults (mean 27 yr) *49 (15/34)*	Weighed Food Diary	1d	4d	Mean Difference; Correlation Coefficient (S); Class Classification; Limits of Agreement
Hartwell and Henry (2001)	Self ≥100 food items / questions	DIET5	Hartwell and Henry (2001)	Energy; Macronutrients (8); Micronutrients (4) Food Group	Adults (mean 45–75 yr) *25 (16/9)*	Estimated Food Diary	2d	8d	Mean Difference; Correlation Coefficient (P);
Heath et al. (2005) (MBIAT)	Interview ≥100 food items / questions	MCW4 & MCW5	Heath et al. (2005)	Micronutrients (4) Food Groups	Adults (46–75 yr) *48 (48/0)*	Weighed Food Diary	3d	12d	Mean Difference; Correlation Coefficient (S); Cross Classification
Heller, Pedoe, and Rose (1981)	Self ≤50 food items / questions	Not Reported	Heller, Pedoe, and Rose (1981)	Macronutrients (1)	Adults (40–59 yr) *68 (68/0)*	Weighed Food Diary	1d	3d	Correlation Coefficient
Hooper et al. (2010)	Self ≥100 food items / questions	MCW6	Hooper et al. (2010)	Energy; Macronutrients (3); Food Groups	Adults (mean 29–55 yr) *263*	24-hour recall	1d	1d	Correlation Coefficient (P)
Kassam-Khamis et al. (1999)	Interview >51–99 food items / questions	COMP-EAT4; data on traditional South Asian foods & MCW5	Kassam-Khamis et al. (1999)	Energy; Macronutrients (4)	Adults (25–50 yr) *11 (0/11)*	Weighed Food Diary	1d	7d	Median Paired Difference; Correlation Coefficient (P); Cross Classification
			Sevak et al. (2004)	Energy; Macronutrients (8); Micronutrients (7)	Adults (34–75 yr) *11 (0/11)*	24-Hour Recall	1d	12 x 1d over 12 months	Individual Means; Correlation Coefficient (P & S); Cross Classification; Cohen’s kappa
Lanham (1993)	Self >51–99 food items / questions	MCW	Bodner et al. (1998)	Micronutrients (4)	Adults (39-45 yr) *273 (118/ 155)*	Biomarkers	1d	1d	Individual Means; Correlation Coefficient (P); Cross Classification
Dong (2003)	Self ≤50 food items / questions	Not Reported	Lean et al. (2003)	Food Groups	Adults (25–64 yr) *1085 (522/ 563)*	FFQ	1d	1d	Median Difference (%); Correlation Coefficient
Little et al. (1999) (HEA1)	Self; Interview >51–99 food items / questions	Royal Society of Cambridge Database	Little et al. (1999)	Micronutrients (1); Food Groups	Adults & Elderly (18-80 yr) *111 (53/58)*	Weighed Food Diary	1d	7d	Median Difference (%); Correlation Coefficient (S)
Little et al. (1999) (HEA2)	Self; Interview >51–99 food items / questions	Royal Society of Cambridge Database	Little et al. (1999)	Micronutrients (1); Food Groups	Adults & Elderly (18–80 yr) *111 (53/58)*	Weighed Food Diary	7d	7d	Median Difference (%); Correlation Coefficient (S)
Little et al. (1999) (HEA3)	Self; Interview >51–99 food items / questions	Royal Society of Cambridge Database	Little et al. (1999)	Micronutrients (1); Food Groups	Adults & Elderly (18–80 yr) *111 (53/58)*	Weighed Food Diary	7d	7d	Median Difference (%); Correlation Coefficient (S)
Little et al. (1999) (Nurse Questions)	Interview >51–99 food items / questions	Royal Society of Cambridge Database	Little et al. (1999)	Micronutrients (1); Food Groups	Adults & Elderly (18–80 yr) *111 (53/58)*	Weighed food diary	1d	7d	Median Difference (%); Correlation Coefficient (S)
Margetts, Cade, and Osmond (1989)	Self >51–99 food items / questions	MCW4	Margetts, Cade, and Osmond (1989)	Energy; Macronutrients (4); Micronutrients (4)	Adults (35–54 yr) *433*	24-hour recall	1d	1d	Correlation Coefficient (S); Cross Classification
Masson et al. (2003) (Scottish Collaborative Group FFQ)	Self ≥100 food items / questions	UK National Nutrient Databank & MCW	Masson et al. (2003)	Energy; Macronutrients (9); Micronutrients (15)	Adults (19–58 yr) *81 (41/40)*	Weighed food diary	1d	4d	Relative Median Difference (%); Correlation Coefficient (S); Cross Classification; Weighed Cohen’s kappa
			Heald et al. (2006)	Energy; Micronutrients (4)	Adults & Elderly (51–75 yr) *203 (203/0)*	Biomarkers	1d	1d	Individual Medians; Correlation Coefficient (S); Cross Classification; Weighed Cohen’s kappa
			Jia et al. (2008)	Energy; Macronutrients (9); Micronutrients (15)	Elderly (64-80 yr) *83 (42/41)*	Weighed food diary	1d	4d	Mean Difference; Correlation Coefficient (S); Cross Classification; Weighed Cohen’s kappa
			Mohd-Shukri et al. (2013)	Energy; Macronutrients (10); Micronutrients (25)	Pregnant Women (21–45 yr) *63 (0/63)*	Weighed Food Diary	1d	4d (Inc. 1 weekend d)	Individual Medians; Correlation Coefficient (P & S); Cross Classification; Weighted Cohen’s kappa.
			Hollis et al. (2017)	Energy; Macronutrients (9); Micronutrients (16); Food Group	Adults (18–65 yr) *96 (40/56)*	Estimated Food Diary	1d	7d consecutive	Mean difference; Correlation Coefficient (S); Cross Classification; Limits of Agreement; Weighted Cohen’s kappa
McKeown et al. (2001) (EPIC FFQ)	Self ≥100 food items / questions	MCW	McKeown et al. (2001)	Energy; Macronutrients (7); Micronutrients (6); Food Groups	Adults & Elderly (45–74 yr) *146 (58/88) = Food Diary; 134 (57/77)=Biomarkers*	Weighed Food Diary; Estimated Food Diary & Biomarkers	1d	7d (Food Diary); 3 × 1d (Biomarkers)	Individual Means; Correlation Coefficient (P & S); Cross Classification
			Day et al. (2001)	Micronutrients (2)	Adults & Elderly (45–74 yr) *123*	Estimated Food Diary & Biomarkers	1d	7d (Food Diary); 6d over 12 months (Biomarkers)	Individual Means; Correlation Coefficient
			Lietz et al. (2002)	Energy; Macronutrients (6); Micronutrients (3)	Adolescents (11.8–13.2 yr) *50 (32/18)*	Weighed Food Diary	1d	7d	Mean Difference; Correlation Coefficient (S); Cross Classification; Limits of Agreement
Mouratidou, Ford, and Fraser (2006)	Self >51–99 food items / questions	MCW5	Mouratidou, Ford, and Fraser (2006)	Energy; Macronutrients(11); Micronutrients (24); Food Groups	Pregnant Women (17–43 yr) *123 (0/123)*	24-Hour Recall	1d	2d	Individual Means; Correlation Coefficient (P); Cross Classification; Limits of Agreement
Nelson et al. (1988)	Interview ≤50 food items / questions	MCW4	Nelson et al. (1988)	Micronutrients	Elderly (65–90 yr) *30 (0/30) = Food Diary; 28 (13/15) =Duplicate Diet*	Weighed Food Diary & Duplicate Diet	2d (vs Food Diary); 1d (vs Duplicate Diet)	7d (Food Diary); 5d (Duplicate Diet)	Individual Means; Correlation Coefficient; Cross Classification
O'Donnell et al. (1991)	Self ≥100 food items / questions	DIET	O'Donnell et al. (1991)	Energy; Macronutrients (4); Micronutrients (14);	Adults (19–65 yr) *52 (24/28)*	Weighed Food Diary & Biomarkers	1d	4 × 4d at 1 month intervals (Food Diary); 4d (Biomarkers)	Individual Means; Correlation Coefficient (P); Class Classification
Papadaki and Scott (2007)	Self ≤50 food items / questions	Not Reported	Papadaki and Scott (2007)	Food Groups	Adults (25–55 yr) *51 (0/51)*	Estimated Food Diary	1d	7d	Individual Means; Correlation Coefficient (P); Cross Classification; Limits of Agreement; Weighted Cohen’s kappa
Pufulete et al. (2002)	Self >51–99 food items / questions	MCW5	Pufulete et al. (2002)	Micronutrients (1)	Adults (22–65 yr) *36 (16/20)*	Weighed Food Diary & Biomarkers	2d	7d	Individual Means; Correlation Coefficient; Cross Classification
Robinson et al. (2007)	By-Proxy ≤50 food items / questions	MCW5	Robinson et al. 2007	Energy; Macronutrients (4); Micronutrients (18)	Infants (6 months) *50 (25/25)*	Weighed Food Diary	1d	4d	Mean Difference (%); Correlation Coefficient (S); Limits of Agreement;
Robinson et al. (2007)	By-Proxy ≤50 food items / questions	MCW5	Robinson et al. 2007	Energy; Macronutrients (4); Micronutrients (18)	Infants (12 months) *50 (27/23)*	Weighed Food Diary	1d	4d	Mean Difference (%); Correlation Coefficient (S); Limits of Agreement;
Roddam et al. (2005)	Self ≤50 food items / questions	MCW5	Roddam et al. (2005)	Energy; Macronutrients (9); Micronutrients (12); Food Groups	Adults (50–64 yr) *202 (0/202)*	Weighed Food Diary & Estimated Food Diary	2d	7d	Median Difference (%); Correlation Coefficient (P); Cross Classification; Weighted Cohen’s kappa
Roe et al. (1994) (DINE)	Interview ≤50 food items / questions	MCW4	Roe et al. (1994)	Energy Macronutrients (4)	Adults (17–62 yr) *206 (128/78)*	Estimated Food Diary	1d	4d	Correlation Coefficient (P); Cross Classification
			Little et al. (1999)	Macronutrients (1); Micronutrients (1); Food Groups	Adults & Elderly (18–80 yr) *111 (53/58)*	Weighed Food Diary	1d	7d	Median Difference (%) Correlation Coefficient (S);
Sofianou-Katsoulis et al. (2011)	By-Proxy ≤50 food items / questions	Not Reported	Sofianou-Katsoulis et al. (2011)	Food Groups	Children (3–7 yr) 33	24-Hour Recall	1d	7d	Individual Means
Venter et al. (2006)	Not reported ≤50 food items / questions	Not Reported	Venter et al. (2006)	Food Groups	Pregnant Women (20-44 yr) *57 (0/57)*	Estimated Food Diary	1d	7d	Cross Classification; Cohen’s kappa
Yarnell et al. (1983)	Self >51–99 food items / questions	MCW4 & MCW5	Thompson and Margetts (1993)	Energy; Macronutrients (9); Micronutrients (6) Food Group	Adults (40–59 yr) *301 (122/ 179)* Smokers only	Biomarkers	1d	10d	Mean Difference; Correlation Coefficient (S); Limits of Agreement
			Bolton-Smith et al. (1991)	Micronutrients (5);	Adults (41–50 yr) *196 (196/0)*	Biomarkers	1d	Not Reported	Individual Means; Correlation Coefficient (P); Cross Classification;
Food Checklist									
Bingham et al. (1994)	Self ≥100 food items / questions	MCW4	Bingham et al. (1994) (pictures & no pictures)	Energy; Macronutrients (7); Micronutrients (6)	Adults (50–65 yr) *160 (0/160)*	Weighed Food Diary	7d	4 × 4d	Individual Means; Correlation Coefficient (S); Cross Classification
			Bingham et al. (1997) [(pictures & no pictures)	Micronutrients (3)	Adults (50–65 yr) *160 (0/160)*	Biomarkers	7d	8d over 12 months	Correlation Coefficient (P & S)
			Little et al. (no pictures) (1999)	Macronutrients (1); Micronutrients (1); Food Groups	Adults & Elderly (18–80 yr) *111 (53/58)*	Weighed Food Diary	7d	7d	Median Difference (%); Correlation Coefficient (S)
			Johansson (2008) (no pictures)	Energy; Macronutrients (6); Micronutrients (6); Food Groups	Elderly (55–88 yr) *80 (80/0)*	Weighed Food Diary	4d	4 × 4d over 12 months	Individual Means
Cade, Frear, and Greenwood (2006) (CADET)	Self; By-Proxy ≥100 food items / questions	DANTE	Cade, Frear, and Greenwood (2006)	Energy; Macronutrients (7); Micronutrients (5); Food Groups	Children (3–7 yr) *180 (100/80)*	Semi-Weighed Food Diary	1d	1d	Mean Difference; Correlation Coefficient (S); Limits of Agreement
			Christian et al. (2015)	Energy; Macronutrients (5); Micronutrients (3); Food Groups	Children (8–11 yr) *67 (33/34)*	Weighed Food Diary	1d	1d	Mean Difference; Correlation Coefficient; Limits of Agreement
Johnson and Hackett (1997) (FIQ)	**Self	Not reported	Johnson et al. (2001)	Food Groups	Adolescents (11–13 yr) *93 (41/52)*	Estimated Food Diary	1d	3d	Correlation Coefficient (P)
Holmes, Dick, and Nelson (2008)	Self; By-Proxy; Interview ≥100 food items / questions	MCW5	Holmes, Dick, and Nelson (2008)	Energy; Macronutrients (4); Micronutrients (6); Food Group	Children, Adolescents, Adults, Elderly (2–90 yr) *76, 48, 206, 54* Low SES	Weighed Food Diary	4d	4d	Mean Difference
Diet History									
Black, Welch, and Bingham (2000)	Interview	MCW4	Black, Welch, and Bingham (2000)	Energy; Macronutrients (2)	Adults (50–65 yr) *64 (0/64)*	Weighed Food Diary; DLW; Biomarkers	1d	4 × 4d over 12 months (Food Diary); 8d over 12 months (Biomarkers); 14d (DLW)	Mean Difference; Correlation Coefficient (P); Limits of Agreement
Livingstone et al. (1992)	By Proxy; Interview	MCW4	Livingstone et al. (1992)	Energy	Children & Adolescents (3–18 yr) *78 (41/37)*	DLW	1d	10-14d	Mean Difference (%); Limits of Agreement
Jackson, Little, and Wilson (1990)	Interview	MCW4	Jackson, Little, and Wilson (1990)	Macronutrients (2); Micronutrients (1)	Elderly (59–74 yr) *80 (39/41)*	FFQ	1d	1d	Individual Means or Medians; Correlation Coefficient (P & S); Cross Classification; Weighted Cohen’s kappa

*Studies that included multiple pass/days recall.

**Tool is web/smartphone based.

MCW = McCance & Widdowson; DLW = Doubly Labeled Water; SES = Socio-economic status.

### Characteristics of the validation studies

A total of 66 validation papers were identified for the 63 DATs. Eight (12%) involved multiple DATs, and 13 (20%) tools were validated in multiple validation papers (Table 3). Five validation studies focused specifically on males (Bolton-Smith et al. 1991; Heath et al. 2005; Heller, Pedoe, and Rose 1981; Johansson 2008; Heald et al. 2006) and 13 on females (e.g. Papadaki and Scott 2007; Mouratidou, Ford, and Fraser 2006).

Of the 63 DATs, 53 (84%) were validated against a different type of dietary assessment method, most of these were weighed food diaries (*n* = 40, 75%), with nine (14%) of the tools using more than one reference method for validation. Four (6%) (Bolton-Smith et al. 1991; McKeown et al. 2001; Yarnell et al. 1983; Lietz et al. 2002) of the 63 tools were exclusively validated against biomarkers, four (6%) (Johnson, Driscoll, and Goran 1996; Livingstone et al. 1992; Davies et al. 1994; Montgomery et al. 2005) against DLW, and two (3%) (Hillier et al. 2012; Edmunds and Ziebland 2002) against direct observation. The sample size of the validation studies varied by type of DAT and the comparator and ranged from 11 to 2265.

Of the 63 DATs, 46 (73%) validated at least one macronutrient, with 36 (57%) validating fat, 31 (49%) carbohydrate, 28 (44%) protein, and 15 (24%) saturated fat with two (3%) tools validating particular types of fat such as fatty acids (Broadfield et al. 2003) and cholesterol (Heller, Pedoe, and Rose 1981). Micronutrients were validated in 46 (73%) tools, with the most frequently measured being vitamin C (*n* = 34, 54%), calcium (*n* = 29, 46%), and iron (*n* = 22, 35%). Four (6%) of the tools validated micronutrients only, with two of these (3%) measuring one micronutrient only (Nelson et al. 1988; Pufulete et al. 2002). Energy was validated in 35 (55%) of the tools with two (3%) of these not validating any other aspect of diet (Livingstone et al. 1992; Davies et al. 1994). At least one food group was validated in 49 (78%) of the tools: 18 (28%) validating fruits, 17 (27%) validating vegetables, and 10 (16%) validating food groups exclusively.

The statistical methods used to compare the difference in measurement between the DAT and reference methods varied with 55 (79%) using correlation coefficients and five (8%) of these not using another statistical method. The mean or median difference (MD) was used by 41 (65%) of the studies while 22 (35%) only published the mean/median of the tool and reference method separately. One (2%) study only used the mean difference (Holmes, Dick, and Nelson 2008). Cross classification (percentage agreement) was used in 33 (51%) studies, LOA in 24 (38%) studies, and Cohen’s Kappa in 10 (16%) studies. Only three (5%) used all five statistical methods with 10 (15%) using four methods.

### Nutritools website to assist researchers to compare and choose DATs

Over 900 validation results covering 5 lifestages, 18 nutrients, 6 dietary assessment, and 9 validation method types were extracted from the 63 validated DATs identified. This information was incorporated into the interactive www.nutritools.org/website developed to help researchers choose tools appropriate for their research question from the on-line library of DATs found from the reviews.

First, researchers are encouraged to follow the Step-by-Step Best Practice Guidelines (BPG) on the website that were developed by expert consensus to help users select the most suitable DAT for their study (Cade et al. 2017, www.nutritools.org/guidelines). These interactive guidelines help researchers filter the list of DATs to show only those in the tool library most appropriate for their research question. Information about strength and weakness of different DAT types are also on the website (www.nutritools.org/strengths-and-weaknesses) along with other helpful information.

Alternatively, a researcher can select DATs that meet criteria of interest to them using the tool and validation method filter from the Dietary Assessment Tool menu (www.nutritools.org/tools) by selecting tool type and validation characteristics. For instance, selecting “Biomarkers” and “Doubly labeled water” to validate energy displays 17 UK DATs validated using these methods. Alternatively, selecting “online” as the Format in the Tool filter displays 12 UK DATs that can be completed online. From the library of tools, the summary plots, or bubble chart menu (www.nutritools.org/tools/visualization), the users are able to view the specific validation results and visually compare the selected DATs. Information about whether validations were on specific populations is also provided.

**Figure 2.**
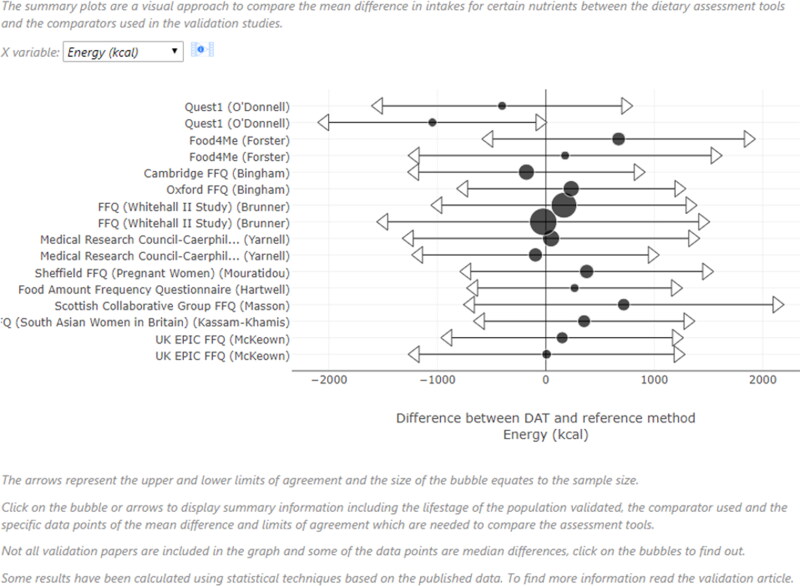
An example of a summary plot on the www.nutritools.org website.

Validation results from different studies can be compared on the website via summary plots, a novel visualization method (www.nutritools.org/tools/summary-plots), selecting from over 500 Bland-Altman limit of agreement validations relating to the 63 UK DATs. For example, using the filters to select FFQs, energy, adults and UK validations, the mean difference (MD) in estimated intakes between the tested DAT and the reference method, and the lower and upper Bland Altman limits of agreement (LOA) (Bland and Altman 1986) for these criteria are displayed in the summary plot observed in Fig. 2. From the filtered results, researchers should avoid choosing a DAT with large mean differences (the central dot on each horizontal line) from the zero line of no difference (e.g. the Quest1 FFQ (O'Donnell et al. 1991) and wide LOA (the distance between arrows at the ends of each result line).

### Mean differences and limits of agreements (LOAs) tabulated by tool and reference type

Table 4 provides a summary of energy and nutrient findings for the validation studies where the lower and upper Bland-Altman LOA were reported in absolute terms or could be calculated from the MD between the reference method and tool along with the standard deviation. There were many gaps in the evidence available, with no evidence for use of doubly labeled water (DLW) as a reference method in adults/elderly and energy intakes. No studies in children used a diary or recall as the reference method for protein intake. There were no biomarker studies reported for calcium, iron, folate, or zinc. Overall there were over 500 separate validations for which LOA could be determined involving different nutrients, age ranges, and/or genders. The majority used a weighed food diary as the reference method, and in adults the majority of these were for validating FFQs or food check lists. DLW was also used to validate energy intake in child’s but not adult studies. Biomarkers were used to validate protein, retinol, vitamin C, and sodium in a small number of adult studies. The results vary substantially depending on the type of tool validated and the reference method used.

**Table 4.** Summary of validation results by reference method type, tool type and nutrient.

Validation reference method / nutrient	Tool type	Number of validation study results^#^		Weighted mean differences*		Range of limits of agreement reported	
		Infants, children and adolescents	Adults and elderly	Infants, children and adolescents	Adults and elderly	Infants, children and adolescents	Adults and elderly
**Energy (kcal)**							
Doubly labeled water	Food diary	3	0	−138	–	−1747 to 1045	–
	Dietary Recall	3	0	70	–	−1102 to 879	–
	FFQ / Food checklist	0	0	–	–	–	–
Food diary	Food diary	5	6	−18	−46	−1259 to 1261	−1223 to 1201
	Dietary Recall	4	9	254	−47	−836 to 1628	−1301 to 1706
	FFQ / Food checklist	7	19	247	52	−1497 to 1912	−2036 to 2129
24-hour recall	Food diary	0	1	–	−52	–	−582 to 483
	Dietary Recall	1	1	−55	3	−797 to 687	−1108 to 1113
	FFQ / Food checklist	0	2	–	366	–	−726 to 1480
FFQ	Food diary	0	0	–	–	–	–
	Dietary Recall	0	0	–	–	–	–
	FFQ / Food checklist	0	1	–	671	–	−523 to 1865
**Protein (g)**							
Biomarker*	Food diary	0	1	–	0.9	–	−5 to 6.8
	Dietary Recall	0	0	–	–	–	–
	FFQ / Food checklist	1	1	8.1	2.3	−3.5 to 19.7	−7 to 12
Food diary	Food diary	5	6	0.2	−2.2	−64 to 61	−75 to 67
	Dietary Recall	4	8	8.4	−0.9	−40 to 61	−67 to 79
	FFQ / Food checklist	7	19	10.1	6.0	−66 to 89	−71 to 68
24-hour recall	Food diary	0	1	–	−4.0	–	−34 to 26
	Dietary Recall	1	1	−2.0	−1.0	−45 to 41	−47 to 45
	FFQ / Food checklist	0	2		11.9		−39 to 70
FFQ	Food diary	0	0	–	–	–	–
	Dietary Recall	0	0	–	–	–	–
	FFQ / Food checklist	0	1	–	−21.0	–	−36 to 78 to 37
**Carbohydrate(g)**							
Food diary	Food diary	5	6	−5.6	−10.9	−185 to 192	−211 to 172
	Dietary Recall	4	8	30.2	−8.7	−132 to 229	−161 to 196
	FFQ / Food checklist	7	19	36.2	18.5	−238 to 305	−240 to 209
24-hour recall	Food diary	0	1	–	−2.0	–	−98 to 94
	Dietary Recall	1	1	−11.0	−5.0	−152 to 130	−149 to 139
	FFQ / Food checklist	0	2	–	35.1	–	−112 to 177
FFQ	Food diary	0	0	–	–	–	–
	Dietary Recall	0	0	–	–	–	–
	FFQ / Food checklist	0	1	–	−85.0	–	−66 to 236
**Total sugars (g)**							
Food diary	Food diary	0	1	–	1.0	–	−45 to 47
	Dietary Recall	0	2	–	0.5	–	−74 to 86
	FFQ / Food checklist	2	14	38.7	12.4	−129 to 200	−114 to 122
24-hour recall	Food diary	0	0	–	–	–	–
	Dietary Recall	1	1	−14.0	−4.0	−121 to 92	−92 to 83
	FFQ / Food checklist	0	1	–	−6.0	–	−86 to 98
FFQ	Food diary	0	0	–	–	–	–
	Dietary Recall	0	0	–	–	–	–
	FFQ / Food checklist	0	1	–	−26.0	–	−42 to 94 to 42
**Fat (g)**							
Food diary	Food diary	5	6	−0.03	1.6	−58 to 64	−51 to 60
	Dietary Recall	4	9	11.8	−0.5	−50 to 88	−71 to 87
	FFQ / Food checklist	7	20	8.6	−4.3	−75 to 99	−99 to 71
24-hour recall	Food diary	0	1	–	−3.0	–	−35 to 29
	Dietary Recall	1	1	−3.0	4.0	−52 to 46	−62 to 69
	FFQ / Food checklist	0	2	–	19.6	–	−39 to 80
FFQ	Food diary	0	0	–	–	–	–
	Dietary Recall	0	0	–	–	–	–
	FFQ / Food checklist	0	1	–	−23.0	–	−32 to 78 to 31
**Dietary fiber (g)**							
Food diary	Food diary	0	2	–	−0.2	–	−8 to 7
	Dietary Recall	0	3	–	−0.1	–	−13 to 17
	FFQ / Food checklist	3	7	2.6	2.5	−19 to 23	−13 to 19
24-hour recall	Food diary	0	0	–	–	–	–
	Dietary Recall	1	1	−1.0	1.0	−10 to 8	−12 to 15
	FFQ / Food checklist	0	2	–	4.8	–	−6 to 19
FFQ	Food diary	0	0	–	–	–	–
	Dietary Recall	0	0	–	–	–	–
	FFQ / Food checklist	0	0	–	–	–	–
**Retinol (µg)**							
Biomarkers	Food diary	0	0	–	–	–	–
	Dietary Recall	0	0	–	–	–	–
	FFQ / Food checklist	0	2	–	121	–	−979 to 1153
Food diary	Food diary	0	2	–	95.1	–	−2084 to 2226
	Dietary Recall	0	2	–	89.0	–	−7360 to 7906
	FFQ / Food checklist	0	8	–	71.9	–	−2410 to 2450
24-hour recall	Food diary	0	0	–	–	–	–
	Dietary Recall	0	0	–	–	–	–
	FFQ / Food checklist	0	1	–	92.4	–	341 to 526
FFQ	Food diary	0	0	–	–	–	–
	Dietary Recall	0	0	–	–	–	–
	FFQ / Food checklist	0	1	–	60.0	–	−425 to 545
**Vitamin C (mg)**							
Biomarkers	Food diary	0	0	–	–	–	–
	Dietary Recall	0	0	–	–	–	–
	FFQ / Food checklist	0	2	–	26.9	–	−32 to 80
Food diary	Food diary	4	6	−2.5	−5.4	−147 to 145	−169 to 155
	Dietary Recall	4	8	16.5	−1.0	−108 to 154	−159 to 197
	FFQ / Food checklist	5	20	16.5	54.9	−168 to 216	−164 to 349
24-hour recall	Food diary	0	0	–	–	–	–
	Dietary Recall	0	1	–	−7.0	–	−202 to 188
	FFQ / Food checklist	0	1	–	−0.7	–	−97 to 96
FFQ	Food diary	0	0	–	–	–	–
	Dietary Recall	0	0	–	–	–	–
	FFQ / Food checklist	0	1	–	57.4	–	−70 to 185
**Calcium (mg)**							
Food diary	Food diary	4	6	8.7	−48.3	−663 to 630	−767 to 597
	Dietary Recall	4	8	87.0	−20.6	−565 to 744	−822 to 873
	FFQ / Food checklist	7	21	76.7	38.0	−673 to 836	−1003 to 1142
24-hour recall	Food diary	0	0	–	–	–	–
	Dietary Recall	0	1	–	−8.8	–	−686 to 668
	FFQ / Food checklist	0	2	–	111	–	−646 to 769
FFQ	Food diary	0	0	–	–	–	–
	Dietary Recall	0	0	–	–	–	–
	FFQ / Food checklist	0	1	–	−324	–	−467 to 1115 to 467
**Iron(mg)**							
Food diary	Food diary	4	6	−0.7	−0.7	−9.6 to 7.2	−10.3 to 8.5
	Dietary Recall	4	8	0.7	−0.1	−6.6 to 9.4	−11.9 to 13.3
	FFQ / Food checklist	5	20	1.1	0.3	−7.7 to 8.0	−14 to 13.4
24-hour recall	Food diary	0	0	–	–	–	–
	Dietary Recall	0	1	–	0.4	–	−9.1 to 9.9
	FFQ / Food checklist	0	2	–	2.5	–	−5.7 to 11.2
FFQ	Food diary	0	0	–	–	–	–
	Dietary Recall	0	0	–	–	–	–
	FFQ / Food checklist	0	1	–	6.2	–	−4 to 17
**Folate (µg)**							
Food diary	Food diary	4	5	−10.7	−17.2	−309 to 259	−497 to 451
	Dietary Recall	4	6	11.3	−6.5	−257 to 263	−307 to 417
	FFQ / Food checklist	5	15	31.4	70.9	−268 to 300	−244 to 336
24-hour recall	Food diary	0	0	–	--	–	–
	Dietary Recall	0	1	–	24.5	–	−214 to 263
	FFQ / Food checklist	0	2	–	48.4	–	−106 to 205
FFQ	Food diary	0	0	–	--	–	–
	Dietary Recall	0	0	–	--	–	–
	FFQ / Food checklist	0	1	–	−125	–	−106 to 356
**Sodium (mg)**							
Biomarker	Food diary	0	1	–	--572	–	−3103 to 1960
	Dietary Recall	0	0	–	–	–	–
	FFQ / Food checklist	0	1	–	−575	–	−3875 to 2725
Food diary	Food diary	0	0	–	–	–	–
	Dietary Recall	0	0	–	–	–	–
	FFQ / Food checklist	2	6	571	−190	−2879 to 3715	−3956 to 2620
24-hour recall	Food diary	0	0	–	–	–	–
	Dietary Recall	1	0	−20.0	–	−2900 to 2900	–
	FFQ / Food checklist	0	1	–	106	–	−2048 to 2260
FFQ	Food diary	0	0	–	–	–	–
	Dietary Recall	0	0	–	–	–	–
	FFQ / Food checklist	0	1	–	−155	–	−1615 to 1926
**Zinc (mg)**							
Food diary	Food diary	0	0	–	–	–	–
	Dietary Recall	0	0	–	–	–	–
	FFQ / Food checklist	0	4	–	1.7	–	−10 to 9
24-hour recall	Food diary	0	0	–	–	–	–
	Dietary Recall	0	0	–	–	–	–
	FFQ / Food checklist	0	1	–	1.6	–	−4 to 7
FFQ	Food diary	0	0	–	–	–	–
	Dietary Recall	0	0	–	–	–	–
	FFQ / Food checklist	0	0	–	–	–	–

*Nitrogen values, not protein values.

^#^Results for different age groups and genders within the two main age groups were taken into account separately.

*Weighted mean differences between the intakes = test tool mean intake minus reference method mean intake; these were weighted using the number of individuals taking part in each validation studies to calculated the overall mean difference for each validation and tool type combination.

For the majority of the 37 WMD of the infant, children and adolescent validations, the DATs showed an over estimation compared to the reference method (*n* = 23 62%), with the adult/elderly studies showing an underestimation for 39 (49%) and an overestimation for 40 (51%) compared to the reference method. The range of LOAs appeared wide in most cases. For example, the WMD in energy for infants/children from a food diary compared to DLW was −138 kcal, with a wide range of LOA from −1747 to 1045. In adults, large mean differences were observed for energy when comparing an FFQ/food checklist against an FFQ (WMD 671, LOA −523 to 1865); however, a wider range of LOAs were observed when comparing FFQ/food checklist against food diaries (WMD 52, LOA −2036 to 2129). In general, when an FFQ/food checklist was the DAT being tested against a comparator, the WMD were larger and LOA wider than for other types of DAT compared against similar reference methods for macronutrients.

## Discussion

To our knowledge, this is the first detailed systematic review of reviews of DATs to identify and collate data on validated DATs. The systematic review identified 63 UK validated DATs. The majority of these DATs were FFQs validated on adults. Results were extracted and incorporated into the interactive www.nutritools.org website; this can guide researchers to search for suitable validated DATs. However, only a small percentage of validation studies used objective validation measures such as biomarkers and only about half of all validations used the Bland-Altman limits of agreement statistical method.

For infants, children, and adolescents, the range of nutrients validated, particularly micronutrients, was much less than for the adult studies. For example, no DAT validating zinc intake in children was found, despite a recognized deficiency among children and adolescents in the UK, particularly females in the 11–18 age bracket (Bates et al. 2014).

The most common type of DAT for assessing dietary intake was the FFQ. FFQs generally aim to collect and capture usual/long-term intake particularly from larger populations, due to their relative low administration cost and low participant burden compared to other tools (Shim, Oh, and Kim 2014; Carroll et al. 2012). However, limitations of FFQs include recall bias, missing data, and under/over-reporting. These are attributed to reliance on participant’s memory, inability to accurately estimate portion sizes and misinterpretation of the questions, or social desirability bias (Poslusna et al. 2009; Thompson and Subar 2008; Satija et al. 2015). Furthermore, choice of FFQ and food checklist length should depend on the overall study aim and whether energy or full nutrient intake is being measured (Thompson et al. 2010). A third of the FFQs in this review were long (≥100 food questions/items), and although higher correlation coefficients in validations have been observed with long FFQs (Livingstone, Robson, and Wallace 2004; Lean et al. 2003), short FFQs can capture a high percentage of nutrient intake when designed to measure specific nutrients (Lean et al. 2003; Bingham 2002).

While food diaries and recalls try to overcome some of the issues of FFQs by collecting current dietary intakes (Thompson and Subar 2008), they also rely on self-reporting, thus having similar limitations, along with a higher respondent burden, which can result in a temporary change during recording from their habitual intake (Poslusna et al. 2009; Thompson and Subar 2008; Satija et al. 2015).

In relation to time frame, FFQs, food checklists, and diet histories provide flexibility to measure dietary intakes over weeks, months, or a year. Participant burden can limit the scope of other dietary methods, such as food diaries and 24 hour recalls, to short-term intake. However, one of the identified food diaries attempted to measure dietary intake over a year through collection of 16 days of recall equally divided into four periods (seasons) (Bingham et al. 1994). It is important to understand the strength and weaknesses of DAT types when choosing a DAT to use in research; more information can be found on the website (www.nutritools.org/strengths-and-weaknesses).

Administration of the DATs assisted by trained interviewers is one technique used to reduce the issue of missing dietary data and improve the precision of intraindividual variation (Serra-Majem et al. 2009). However, only a few DATs were administered by interviewers due to the time taken and associated expense (Thompson et al. 2010). With the rise in computer and smartphone use, web-based DATs are becoming more popular in nutritional research compared with the traditional pen and paper approach (Carter et al. 2015). New technology can reduce participant and researcher burden, increase adherence, improve data analysis, and reduce the time and cost required for data entry and data coding (Thompson et al. 2010; Hongu et al. 2011; Shriver, Roman-Shriver, and Long 2010); however, paper-based tools were predominant in this review. Limitations of self-reported DATs have led to the development of image-based DATs which can improve the accuracy of measuring dietary intake, due to improvements in portion size estimations limiting misreporting errors (Gemming, Utter, and Mhurchu 2015; O'Loughlin et al. 2013; Gemming et al. 2013). However, issues with these methods can occur, such as procedures not being followed properly, poor image quality, challenges identifying composite dishes, and users forgetting to capture images (Gemming, Utter, and Mhurchu 2015; Rollo et al. 2016). Some of the validated dietary recalls identified were web based, which allows for more complete food databases to be included, supporting users to choose more specific food items. However, this should be achieved without increasing participant burden.

Using an appropriate method to validate a DAT is important (Livingstone, Robson, and Wallace 2004). Due to the difficulty of measuring absolute validity of dietary intake, studies typically measure relative validity, which includes errors associated with the reference method. Most of the tools identified had been tested for relative validity, as the most common reference method used was another self-reported DAT; this has limitations because it is susceptible to similar errors as the tool being validated. Ideally, objective methods such as biomarkers should be used to validate DATs as they are not prone to the self-reporting or bias associated with other reference methods (Bingham 2002; Hedrick et al. 2012). However, these methods only cover a limited number of dietary components and can be expensive and impractical when conducting a large study (Thompson et al. 2010; Hedrick et al. 2012; Freedman et al. 2014). In the present review, only 17 tools were compared against biomarkers, some exclusively and some with additional reference methods. Additionally, the reference method should ideally take into account factors such as seasonality and variation between weekdays and weekends. Generally, this was seen when food diaries and dietary recalls were being validated but not FFQs.

The most common statistical method reported in the validation studies was the correlation coefficient. The use of correlation coefficient as the sole test has been criticized, since it only assesses whether an individual has preserved their ranking in relation to other participants and does not measure absolute agreement (Poslusna et al. 2009; Bland and Altman 1986). However, as FFQs are not necessarily measuring absolute intakes, others have stated this criticism does not apply (Masson et al. 2003). Lombard et al. (2015) argue that a number of statistical approaches should be used in dietary validation studies, however, typically only one to three methods are used out of a possible six (correlation coefficient, paired *t*-test/Wilcoxon signed rank test, percent difference, cross-classification, weighted kappa, Bland-Altman LOA). Ideally, validation studies should include LOA or intra-class correlations (ICC) which measure agreement between a DAT and the reference method, as well as the extent of relative bias in the form of the MD (Bland and Altman 1986). Given this, only results of validation studies that reported the LOA or where this could be calculated in addition to the mean difference were included in our tabulated analysis. Similarly, comparing mean differences and LOAs in the summary plots are the focus on the www.nutritools.org/website to help researchers select DATs. Although researchers may be advised to select DATs with small mean differences and narrow LOAs (or at least avoid those with larger mean differences and wide LOAs), further guidance is needed on what may be classed as small/narrow or large/wide, for instance expressed as a percentage of mean intakes of the population of interest, and/or as absolute values in units of the nutrient.

As observed from the range of the LOA, the estimated intakes can vary widely depending on the tool type and reference method used. The validation method can affect results for particular nutrients resulting in wider LOA. For example, assessing energy intake in children using a weighed food diary can be problematic due to reliance on proxy information from parents and/or carers (Lanigan et al. 2001). Limits of agreement were wide in a study validating a food diary against an FFQ (Broadfield et al. 2003), possibly partly due to limited frequency of consumption options and limited food lists in an FFQ tool. Accurate estimation of the Bland-Altman LOA between two methods can also be compromised by sample size. Studies with a sample size of ≥50 will enable greater accuracy of estimation for particular nutrients (Cade et al. 2002) with ≥100 subjects required to estimate true energy intakes to within 4% of a reference method (Day et al. 2001).

The variation and lack of statistical methods used in validation studies raises concerns about the quality of reporting in nutritional epidemiology. Missing and poor quality description of the validation methodology was found. Lack of information on the development of the DAT was common as a number of tools, especially those which had been adapted from previously developed tools, provided incorrect citations of the methodology papers, noted in other dietary assessment reviews (Bryant et al. 2014). The issues surrounding the variation and the quality of reporting can make recommending one DAT over another difficult (England et al. 2015). To improve the quality of reporting in nutritional epidemiology and dietary assessment research, new guidelines have been developed by the STROBE-nut consortium (Lachat et al. 2016). It is important that these guidelines are promoted, as a higher quality of reporting will allow for easier comparison and understanding of DATs. Additionally, validation study results are not necessarily representative of wider populations. For instance, some validations used or excluded specific populations, which can hinder comparison and selection of DATs. Furthermore, volunteer sampling was the method used by the majority of validation studies through contact via GP surgery, school letters or posters, and/or email advertisements.

### Study strengths and limitations of study

The systematic and comprehensive approach adopted for this study was a strength as it was a practical way of obtaining information on DATs compared to undertaking multiple reviews of each type of DAT for different foods and/or nutrients which would have taken too long given available resources. Cross checking against DAT registers minimized the likelihood of missing tools. Another strength is the interactive nature of the website designed to search and display information about the DATs and their validations, which guides researchers to select appropriate DATs.

The main limitation of this study was that identification of all DATs validated in UK populations could not be guaranteed, as not all of them would have been included in a systematic or literature review. All of these tools are reported in detail on the Nutritools website plus detail on 66 international tools (not discussed in this article). Also despite the date restriction on the published reviews (≥January 2000), there was no date restriction on the actual DAT raising the question of whether tools developed over 25–30 years ago are still fit for purpose today. Additionally, the website will need maintaining to ensure it remains current, holding information on up-to-date tools, including those from other countries and cultures; however limited funds for this are available.

## Conclusions and recommendations

This review identified 63 validated UK DATs which covered a wide range of life stages and nutrients and collated information from these. The characteristics of these DATs, their validation studies, and the validation results are now on the interactive www.nutritools.org website. This can guide researchers to compare and choose the most suitable DAT for their research question, potentially leading to improvement of research in nutritional epidemiology.

This research provides knowledge to assist dietary assessment, having a positive impact on public health policy and society through the potential to support dietary advice and recommendations which can reduce the financial burden of noncommunicable disease.

## Supplementary Material

Supplemental Material

## Appendix 1

1 exp diet/

2 Nutritional status.mp.

3 diet* adj2 intake*.mp

4 diet* adj2 qualit*.mp.

5 food adj2 intake*.mp.

6 nutri* adj2 intake*.mp.

7 diet* adj2 habit*.mp.

8 food adj2 habit.mp.

9 diet* pattern* or meal pattern*.mp.

10 food group*.mp.

11 nutrient*.mp.

12 macro-nutrient* or macronutrient.mp.

13 micro-nutrient or micronutrient.mp.

14 energy intake*.mp.

15 1 or 2 or 3 or 4 or 5 or 6 or 7 or 8 or 9 or 10 or 11 or 12 or 13 or 14

16 diet* adj2 (method* or tool* or survey* or record* or assess*).mp.

17 diet* adj2 (recall* or questionnaire* or histor* or instrument*).mp.

18 nutrition* adj2 (survey* or assess* or instrument*).mp. (27252)

19 food adj2 (questionnaire* or record* or recall* or diar* or checklist* or screener*).mp

20 24* adj2 recall.mp.

21 multiple pass.mp

22 FFQ*.mp

23 diet* adj2 (measure* or analys*).mp

24 nutri* adj2 measur*.mp

25 16 or 17 or 18 or 19 or 20 or 21 or 22 or 23 or 24

26 valid*.mp.

27 reliab*.mp.

28 reproduc*.mp.

29 calibrat*.mp.

30 repeatab*.mp

31 feasib*.mp

32 evaluat*.mp

33 26 or 27 or 28 or 29 or 30 or 31

34 review*.mp

35 meta-analy*.mp.

36 search*.mp.

37 systematic* adj2 (approach or analys*).mp.

38 33 or 34 or 35 or 36

39 15 and 25 and 32 and 37
