# Supplementary material for: A systematic review of reviews identifying UK validated dietary assessment tools for inclusion on an interactive guided website for researchers: www.nutritools.org
Source: Crit Rev Food Sci Nutr. 2019 Mar 18;60(8):1265–89. doi: 10.1080/10408398.2019.1566207 (PMC7114915; doi:10.1080/10408398.2019.1566207)
Supplement: Supplemental Material [file BFSN_A_1566207_SM0939.docx]

# A systematic review of reviews identifying UK validated dietary assessment tools for inclusion on an interactive guided website for researchers: [www.nutritools.org](http://www.nutritools.org)

**Jozef Hooson (JZH)^1^, Jayne Hutchinson (JYH)^1^, Marisol Warthon-Medina^1, 9^, Neil Hancock^1^, Katharine Greathead^1^, Bethany Knowles^1^, Elisa Vargas-Garcia^1^, Lauren E. Gibson^1^, Linda A. Bush^1^, Barrie Margetts^2^, Sian Robinson^3, 4^, Andy Ness^5^, Nisreen A. Alwan^4,6^, Petra A. Wark^7,8^ , Mark Roe^9,10^, Paul Finglas^9^, Toni Steer^11^, Polly Page^11^, Laura Johnson^12^, Katharine Roberts^13,14^, Birdem Amoutzopoulos^11^, Victoria J. Burley^1^, Darren C. Greenwood^15^, Janet E. Cade^1*^ on behalf of the DIET@NET consortium**

*^1^Nutritional Epidemiology Group, School of Food Science and Nutrition, University of Leeds, Leeds, LS2 9JT, UK.*

*^2^Faculty of Medicine, University of Southampton, Southampton SO17 1BJ, UK.*

*^3^MRC Lifecourse Epidemiology Unit, University of Southampton, Southampton SO16 6YD, UK.*

*^4^NIHR Southampton Biomedical Research Centre, University of Southampton & University Hospital Southampton NHS Foundation Trust, Southampton SO16 6YD, UK.*

*^5^*NIHR Biomedical Research Unit in Nutrition, Diet and Lifestyle, University Hospitals Bristol NHS Foundation Trust and the University of Bristol, BS8 1TH, UK.

^6^Academic Unit of Primary Care and Population Sciences, Faculty of Medicine, University of Southampton, Southampton General Hospital, Southampton, SO16 6YD, UK.

^7^Centre for Innovative Research Across the Life Course (CIRAL), Faculty of Health and Life Sciences, Coventry University, Coventry CV1 5FB, UK.

^8^Global eHealth Unit, Department of Primary Care and Public Health, Imperial College London, London SW7 2AZ, UK.

^9^Food Databanks National Capability, Quadram Institute Bioscience, Norwich, NR4 7UA, UK.

^10^EuroFIR AISBL, 40 Rue Washington, 1050 Brussels, Belgium.

^11^MRC Elsie Widdowson Laboratory, Cambridge, CB1 9NL, UK.

^12^Centre for Exercise, Nutrition and Health Sciences, School for Policy Studies, University of Bristol, Bristol, BS8 1TH, UK.

^13^Public Health Section, School of Health and Related Research (ScHARR), University of Sheffield, Sheffield S10 2TN, UK.

^14^Public Health England, London, SE1 8UG, UK.

^15^Faculty of Medicine and Health, Division of Biostatistics, University of Leeds, Leeds LS2 9JT, UK.

*Corresponding author

E-mail: [J.E.Cade@leeds.ac.uk](mailto:J.E.Cade@leeds.ac.uk)

**Abstract**

Background: Health researchers may struggle to choose suitable validated dietary assessment tools (DATs) for their target population. The aim of this review was to identify and collate information on validated UK DATs and validation studies for inclusion on a website to support researchers to choose appropriate DATs.

Design: a systematic review of reviews of DATs was undertaken, DATs validated in UK populations were extracted from the studies identified . A searchable website was designed to display this data. Additionally, mean differences and limits of agreement between test and comparison methods were summarised by method, weighting by sample size.

Results: Over 900 validation results covering 5 life-stages, 18 nutrients, 6 dietary assessment methods and 9 validation method types were extracted from 63 validated DATs which were identified from 68 reviews. These were incorporated into www.nutritools.org. Limits of Agreement were determined for about half of validations. 34 DATs were FFQs. Only 17 DATs were validated against biomarkers, and only 19 DATs were validated in infant/children/adolescents.

Conclusions: The interactive [www.nutritools.org](http://www.nutritools.org) website holds extensive validation data identified from this review and can be used to guide researchers to critically compare and choose a suitable DAT for their research question, leading to improvement of nutritional epidemiology research.

Keywords: Validation studies, Diet records, Systematic Review, Study Characteristics, Dietary Assessment, Limits of Agreement

# Introduction

Diets high in energy dense and nutrient-poor foods have been linked to an increased risk of chronic diseases such as obesity, cardiovascular disease and particular cancers (Rollo et al. 2016). Measuring dietary intake accurately is therefore essential in establishing relationships between food consumption patterns and non-communicable diseases (Serra-Majem et al. 2009); or when evaluating the effectiveness of public health policies and interventions (Mouratidou et al. 2012, Øverby et al. 2009). Accurate measurement of dietary intake, both at an individual and population level is challenging due to measurement difficulties, low participation rates and degree of compliance, with no single method being identified as the best approach for population studies (Shim and Oh, 2014).

Dietary measurement has relied on self-reported dietary assessment tools (DATs) such as food frequency questionnaires (FFQs), 24-hour recalls and weighed/estimated food diaries (WFD, EFD) (Johnson, 2002, Long et al. 2010). However, these methods are prone to selective underreporting, misreporting, are expensive and may have low compliance (Shim and Oh, 2014, Bingham and Day, 1997). Advancements in computer technology have helped address some of these issues (Cade, 2017, Timon et al. 2016). However, it has been recognised that there is no universal DAT which is suitable for all dietary assessment research. A description of the main DATs used to assess dietary intake is shown in supplementary table 1.

A number of key factors should be considered when selecting the most suitable DAT, including the dietary component of interest, the characteristics of the population, the time frame required, the type and accuracy of data required, the food composition table used and the resources available (Cade, 2017). The tool should also be validated for the foods or nutrients of interest and in the population being measured. However, validation information may not be readily available to researchers and not all DATs are easily accessible for use.

The aim of this review was to identify and collate characteristics of DATs which have been validated in the UK population and to include this information together with characteristics of their validation studies and the validation results on the DIET@NET partnership project’s [www.nutritools.org](http://www.nutritools.org) website. The aim of the website is to help researchers and health professionals critically compare and select the most suitable validated DATs for their research question which ultimately may lead to improvements in nutritional epidemiology research. An additional aim was to tabulate the validation results in this manuscript to explore whether they varied by DAT type and reference method type.

# Methodology

A systematic review of reviews of DATs was undertaken to identify validated DATs. Literature reviews as well as systematic reviews were examined, as it was acknowledged that not all validated DATs would be identified through systematic reviews only. From the identified reviews, details of the associated development and validation papers for the UK specific tools were extracted. An unpublished protocol was designed and agreed upon by members of the DIET@NET project.

Search Strategy

To identify reviews of validated DATs the following bibliographic databases were searched: Cochrane Database of Systematic Reviews (CDSR); Database of Abstracts of Reviews of Effectiveness (DARE); National Health Service Economic Evaluation Database (NHS EED); Health Technology Assessment Database (HTA); Web of Science Core Collection; Ovid MEDLINE; In-Process; EMBASE; Scopus; CAB abstracts and Open Grey. The search was initially conducted in May / June 2015, then updated in October 2016 and was restricted to reviews published between January 2000 to October 2016. No restriction was placed on when the tool was developed or validated. Reference lists of the selected reviews and relevant published conference proceedings were also searched. The search-strategy for MEDLINE is shown in Appendix 1. The search-strategy was adapted for other databases when Medical Subject Headings terms were unavailable. Citations were catalogued and managed within Endnote (X7).

Selection of Reviews

Two reviewers (JZH; KG) were independently involved in two rounds of screening to identify reviews that met the eligibility criteria. The first round of screening involved reviewing each article based on their title and abstract. Full copies of potential articles from the previous round were then downloaded for examination by both reviewers independently, to determine eligibility based on the inclusion and exclusion criteria. Any discrepancies between reviewers were reassessed and resolved by further discussion and advice from members of the Diet@Net project board.

Tool identification from reviews

Papers relating to the original DAT development and/or validations identified in the reviews were downloaded and screened to determine eligibility for data extraction (BK). In order to be eligible for this stage of the review, the tools had to satisfy the inclusion criteria. The inclusion and exclusion criteria applied for both the reviews and DATs is noted in Table 1. No date restriction was imposed on the actual tools or their developmental / validation papers. Online searches were carried out for each tool identified for further development or validation papers to ensure all relevant data was collected.

Cross Checking with other sources

It was acknowledged that not all UK validated DATs would be captured by our search strategy, as not all tools may have been included in a review published within the search years (2000-2016). This would particularly disadvantage more recent tools. Therefore, one reviewer (BK) cross checked against DAT registries which were: The National Collaborative on Childhood Obesity Research (NCCOR) and the National Cancer Institute (NCI): Dietary Assessment Primer (Dietary Assessment Calibration/Validation Register: ‘Find a Study’). The Medical Research Council (MRC) website was checked for funded research on diet identifying particular DATs used, along with analysing DATs from MRC funded cohort studies.

Data extraction from the developmental and validation papers and incorporation into website

Two researchers (JH; BK) extracted and collated data from the development and validation papers of the DATs in an Access database and 10% was checked by a third investigator (KG). This data included characteristics of the DATs including lifestage of tool focus; how the tool was administered (by self, proxy or interview) and nutrient database used. Data on the DAT validation studies was also extracted, including the reference method used (eg. 24h recall, weighed food diary, biomarkers and doubly-labelled water) and time span of assessment. Results for validation of energy and 16 nutrients (total fat, saturated fat, monounsaturated fat, polyunsaturated fat, carbohydrate, protein, sugar, fibre (NSP), sodium, calcium, iron, zinc, retinol, folate, vitamin C, vitamin B12) plus fruit and vegetables were extracted. The validation results comparing intakes estimated by the DAT and a reference method for the following statistical methods were extracted where available: mean difference and standard deviation, correlation coefficient, Cohen’s kappa coefficient, percentage agreement and Bland-Altman lower and upper limits of agreement. This data was then incorporated into the website [www.nutritools.org](http://www.nutritools.org). This website was designed and created by Xlab ([www.x-labsystems.co.uk](http://www.x-labsystems.co.uk)) based in Leeds, in collaboration with the Diet@Net team.

Statistical analysis

Data was analysed using Stata version 14 exploring the validation results by DAT and reference method type for energy and selected micro and macro-nutrients to determine whether the validation results varied greatly by type of DAT or by reference method, and to show the number of validations by life stage and nutrient. For this the weighted mean of the differences in intakes (WMD) for each type was calculated, with larger samples having more influence on these summary results.

First the difference in the estimated nutrient intakes from each validation study was determined as the reference method value subtracted from the test DAT. Then the number of individuals taking part in the validation studies were used to produce a weighted mean of these differences by tool and reference method type. Additionally, for each combination of reference method and tool, the range of the lower and upper Bland Altman limits of agreement (LOA) (Bland and Altman, 1986) reported or calculated using the mean difference (MD) and standard deviations from the validation papers, was determined. We summarised these by three types of tools: food diary; 24h dietary recall; FFQ/Food checklist, as these were the most common DAT types used. Diet Histories were not included as there were only a small number of these and they are not commonly used in the UK. These were cross tabulated with four groups of reference measures: recovery biomarkers; food diary; 24-hour recall; FFQ. The results are displayed by two main life stages (i) infants, children and adolescents and ii) adults and elderly.

# Results

A total of 8413 review articles were identified from the database searches (see figure 1). A further seven reviews were identified through reference tracking and internet searches. After removing duplications, 4433 articles remained, with 4297 excluded after screening of the title and abstract. After screening the full texts of the 136 articles, 68 reviews remained of which 29 (43%) were systematic and 39 (57%) were non-systematic literature reviews. No review only reported tools that had been validated in a UK population. The main objective of the reviews varied, with some identifying tools validated for a specific population or life stage, and others focussing on nutrient/food type. The characteristics of the reviews are shown in the supplementary Table 2.

From the reviews, 2972 articles were extracted and screened. Only 169 (6%) of 2972 articles included a UK DAT that measured some aspect of diet, and 99 (59%) of these were excluded after full text screening (see figure 1 for reasons). From these 70 remaining articles, 51 different UK validated DATs were identified, with the review by Cade et al. 2004, providing the most with 24 (46%) validated DATs. Cross checking against DAT registries identified seven additional DATs with a further five identified from internet searching and reference checking making a total of 63 DATs.

Characteristics of the 63 DATs

Out of the 63 DATs, 39 had macro and micronutrient intakes validated in adult and/or elderly populations with a further five validated on all ages, and 19 DATs validated on infants/children and/or adolescents. Ten DATs focussed only on food group intakes (5 adults/elderly only; 1 all ages; 4 infants/children and/or adolescents only). The majority of DATs validated on adults were FFQ, whereas those validated on children and adolescents were food checklists, diaries or 24-hour recalls. The total number and description of the DATs for each separate life stage are shown in Table 2. 12 (19%) of the 63 DATs were a modified version of a previously developed tool (Ashfield-Watt et al. 2007, Broadfield et al. 2003, Bingham et al. 1994, Bodner et al. 1998, Bolton-Smith et al. 1991, Brunner, Junega and Marmot, 2001, Heath et al. 2005, Hillier et al. 2012, Johnson, Driscoll and Goran, 1996, Mouratidou, Ford and Frazer, 2006, Mckeown et al. 2001, Hooper et al. 2010), while the year the 63 DATs were developed ranged from 1981 to 2016.

The DAT characteristics are displayed in Table 3 along with their validation study characteristics; this information can also be found on the interactive website [www.nutritools.org](http://www.nutritools.org). The length of the 34 FFQs ranged from 8-630 food items/questions, with 13 (38%) of these classified as short FFQs consisting of ≤50 food questions / items and 10 (29%) classified as long FFQs consisting of >100 food questions / items. Out of the 63 DATs, 16 (25%) were web based tools by life stage and nutrient. Four tools focussed on infants and toddlers (Lanigan et al, 2001, Marriott et al. 2009, Marriott et al. 2008, Davies et al. 1994). Twelve tools focussed on children and ten tools on adolescents. Forty-seven tools were developed to measure adult diet and 19 were suitable for measuring diet in the elderly. The time frame covered by the DATs varied. Food diaries ranged from measuring intake over one day to repeated measures over one year. Most 24-hour recalls measured the previous 24-hours, however some measured intakes over two consecutive or several days (for example: Johansson, 2008, Hillier et al, 2012, Johnson, Driscoll and Goran, 1996). FFQs ranged from the previous day to usual intake over the previous year with 11 (32%) measuring long term intake (>6 months) and six (16%) measuring short term intake (one day) (Ashfield-Watt et al. 2007, Bingham et al, 1994, Bingham and Day, 1997, Broadfield et al. 2003, Brunner, Juneja and Marmot, 2001, Cleghorn et al. 2016). The food database underpinning the DATs was primarily a version of the McCance and Widdowson’s the Composition of Foods (MCW) food tables or a database based upon MCW. Of the DATs, 10 (16%) did not report the food database used; seven (70%) of these were FFQs.

Characteristics of the validation studies

A total of 66 validation papers were identified for the 63 DATs. Eight (12%) involved multiple DATs and 13 (20%) tools were validated in multiple validation papers (Table 3). Five validation studies focused specifically on males (Bolton-Smith et al. 1991, Heath et al. 2005, Heller, Pedoe and Rose, 1981, Johansson, 2008, Heald et al. 2006) and 13 on females (for example: Papadiki and Scott, 2007, Mouratidou, Ford and Fraser, 2006).

Of the 63 DATs, 53 (84%) were validated against a different type of dietary assessment method, most of these were weighed food diaries (n=40, 75%), with nine (14%) of the tools using more than one reference method for validation. Four (6%) (Bolton-Smith et al. 1991, McKeown et al. 2001, Yarnell et al. 1983, Lietz et al. 2002) of the 63 tools were exclusively validated against biomarkers, four (6%) (Johnson, Driscoll and Goran, 1996, Livingstone et al. 1992, Davies et al. 1994, Montgomery et al. 2005) against DLW and two (3%) (Hillier et al. 2012, Edmunds and Ziebland, 2002) against direct observation. The sample size of the validation studies varied by type of DAT and the comparator and ranged from 11 to 2265.

Out of the 63 DATs, 46 (73%) validated at least one macronutrient, with 36 (57%) validating fat, 31 (49%) carbohydrate, 28 (44%) protein and 15 (24%) saturated fat with two (3%) tools validating particular types of fat such as fatty acids (Broadfield et al. 2003) and cholesterol (Heller, Pedoe and Rose, 1981). Micronutrients were validated in 46 (73%) tools, with the most frequently measured being vitamin C (n=34, 54%), calcium (n=29, 46%) and iron (n=22, 35%). Four (6%) of the tools validated micronutrients only, with two of these (3%) measuring one micronutrient only (Nelson et al. 1988, Pufulete et al. 2002). Energy was validated in 35 (55%) of the tools with two (3%) of these not validating any other aspect of diet (Livingstone et al. 1992, Davies et al. 1994). At least one food group was validated in 49 (78%) of the tools: 18 (28%) validating fruits, 17 (27%) validating vegetables and 10 (16%) validating food groups exclusively.

The statistical methods used to compare the difference in measurement between the DAT and reference methods varied with 55 (79%) using correlation coefficients and five (8%) of these not using another statistical method. Mean or median difference (MD) was used by 41 (65%) of the studies while 22 (35%) only published the mean/median of the tool and reference method separately. One (2%) study only used the mean difference (Holmes, Dick and Nelson, 2008). Cross classification (percentage agreement) was used in 33 (51%) studies, LOA in 24 (38%) studies and Cohens Kappa in 10 (16%) studies. Only three (5%) used all five statistical methods with 10 (15%) using four methods.

Nutritools website to assist researchers to compare and choose DATs

Over 900 validation results covering 5 life-stages, 18 nutrients, 6 dietary assessment and 9 validation method types were extracted from the 63 validated DATs identified. This information was incorporated into the interactive [www.nutritools.org/](http://www.nutritools.org/) website developed to help researchers choose tools appropriate for their research question from the on-line library of DATs found from the reviews.

First, researchers are encouraged to follow the Step-by-Step Best Practice Guidelines (BPG) on the website that were developed by expert consensus to help users select the most suitable DAT for their study (Cade et al. 2017, [www.nutritools.org/guidelines](http://www.nutritools.org/guidelines)). These interactive guidelines help researchers filter the list of DATs to show only those in the tool library most appropriate for their research question. Information about strength and weakness of different DAT types are also on the website (www.nutritools.org/strengths-and-weaknesses) along with other helpful information.

Alternatively a researcher can select DATs that meet criteria of interest to them using the tool and validation method filter from the Dietary Assessment Tool menu ([www.nutritools.org/tools](http://www.nutritools.org/tools)) by selecting tool type and validation characteristics. For instance, selecting “Biomarkers” and “Doubly labelled water” to validate energy, displays 17 UK DATs validated using these methods. Alternatively selecting “online” as the Format in the Tool filter displays 12 UK DATs that can be completed online. From the library of tools, the summary plots or bubble chart menu ([www.nutritools.org/tools/visualisation](http://www.nutritools.org/tools/visualisation)), the users are able to view the specific validation results and visually compare the selected DATs. Information about whether validations were on specific populations is also provided.

Validation results from different studies can be compared on the website via summary plots, a novel visualization method ([www.nutritools.org/tools/summary-plots](http://www.nutritools.org/tools/summary-plots)), selecting from over 500 Bland-Altman limit of agreement validations relating to the 63 UK DATs. For example, using the filters to select FFQs, energy, adults and UK validations, the mean difference (MD) in estimated intakes between the tested DAT and the reference method, and the lower and upper Bland Altman limits of agreement (LOA) (Bland and Altman, 1986) for these criteria are displayed in the summary plot observed in figure 2. From the filtered results, researchers should avoid choosing a DAT with large mean differences (the central dot on each horizontal line) from the zero line of no difference (e.g. the Quest1 FFQ (O’Donell et al. 1991) and wide LOA (the distance between arrows at the ends of each result line).

Mean differences and Limits of agreements (LOAs) tabulated by tool and reference type

Table 4 provides a summary of energy and nutrient findings for the validation studies where the lower and upper Bland-Altman LOA were reported in absolute terms or could be calculated from the MD between the reference method and tool along with the standard deviation. There were many gaps in the evidence available, with no evidence for use of doubly-labelled water (DLW) as a reference method in adults/elderly and energy intakes. No studies in children used a diary or recall as the reference method for protein intake. There were no biomarker studies reported for calcium, iron, folate or zinc. Overall there were over 500 separate validations for which LOA could be determined involving different nutrients, age ranges and/or genders. The majority used a weighed food diary as the reference method, and in adults the majority of these were for validating FFQs or food check lists. DLW was also used to validate energy intake in child’s but not adult studies. Biomarkers were used to validate protein, retinol, vitamin C and sodium in a small number of adult studies. The results vary substantially depending on the type of tool validated and the reference method used.

For the majority of the 37 WMD of the infant, children and adolescent validations, the DATs showed an over estimation compared to the reference method (n=23 62%), with the adult / elderly studies showing an underestimation for 39 (49%) and an overestimation for 40 (51%) compared to the reference method. The range of LOAs appeared wide in most cases. For example, the WMD in energy for infants/children from a food diary compared to DLW was -138kcal, with a wide range of LOA from -1747 to 1045. In adults, large mean differences were observed for energy when comparing an FFQ/food checklist against an FFQ (WMD 671, LOA -523 to 1865), however a wider range of LOAs were observed when comparing FFQ/food checklist against food diaries (WMD 52, LOA -2036 to 2129). In general, when an FFQ/food checklist was the DAT being tested against a comparator, the WMD were larger and LOA wider than for other types of DAT compared against similar reference methods for macronutrients.

# Discussion

To our knowledge this is the first detailed systematic review of reviews of DATs to identify and collate data on validated DATs. The systematic review identified 63 UK validated DATs. The majority of these DATs were FFQs validated on adults. Results were extracted and incorporated into the interactive www.nutritools.org website; this can guide researchers to search for suitable validated DATs. However only a small percentage of validation studies used objective validation measures such as biomarkers and only about half of all validations used the Bland-Altman limits of agreement statistical method.

For infants, children and adolescents the range of nutrients validated, particularly micronutrients was much less than for the adult studies. For example no DAT validating zinc intake in children was found, despite a recognised deficiency amongst children and adolescents in the UK, particularly females in the 11-18 age bracket (Bates et al. 2014).

The most common type of DAT for assessing dietary intake was the FFQ. FFQs generally aim to collect and capture usual / long-term intake particularly from larger populations, due to their relative low administration cost and low participant burden compared to other tools(Shim and Oh, 2014, Carrol et al. 2012). However, limitations of FFQs include recall bias, missing data and under / over-reporting. These are attributed to reliance on participant’s memory, inability to accurately estimate portion sizes and misinterpretation of the questions, or social desirability bias (Poslusna et al. 2009, Thompson and Subar, 2008, Satija et al. 2015). Furthermore, choice of FFQ and food checklist length should depend on the overall study aim and whether energy or full nutrient intake is being measured (Thompson et al. 2010). A third of the FFQs in this review were long (≥100 food questions / items), and although higher correlation coefficients in validations have been observed with long FFQs (Livingstone, Robson and Wallace, 2004, Lean et al. 2003), short FFQs can capture a high percentage of nutrient intake when designed to measure specific nutrients (Lean et al. 2003, Bingham, 2002).

Whilst food diaries and recalls try to overcome some of the issues of FFQs by collecting current dietary intakes (Thompson and Subar, 2008) they also rely on self-reporting, thus having similar limitations, along with a higher respondent burden, which can result in a temporary change during recording from their habitual intake (Poslusna et al. 2009, Thompson and Subar, 2008, Satija et al. 2015).

In relation to time frame, FFQs, food checklists and diet histories provide flexibility to measure dietary intakes over weeks, months or a year. Participant burden can limit the scope of other dietary methods, such as food diaries and 24 hour recalls, to short-term intake. However, one of the identified food diaries attempted to measure dietary intake over a year through collection of 16 days of recall equally divided into four periods (seasons) (Bingham et al. 1994). It is important to understand the strength and weaknesses of DAT types when choosing a DAT to use in research; more information can be found on the website ([www.nutritools.org/strengths-and-weaknesses](http://www.nutritools.org/strengths-and-weaknesses) ).

Administration of the DATs assisted by trained interviewers is one technique used to reduce the issue of missing dietary data and improve the precision of intra-individual variation (Serra-Majem et al. 2009). However, only a few DATs were administered by interviewers due to the time taken and associated expense (Thompson et al. 2010). With the rise in computer and smart-phone use, web-based DATs are becoming more popular in nutritional research compared with the traditional pen and paper approach (Carter et al. 2015). New technology can reduce participant and researcher burden, increase adherence, improve data analysis and reduce the time and cost required for data entry and data coding (Thompson et al. 2010, Hongu et al. 2011, Shriver et al. 2010); however paper-based tools were predominant in this review. Limitations of self-reported DATs has led to the development of image-based DATs which can improve the accuracy of measuring dietary intake, due to improvements in portion size estimations limiting misreporting errors (Gemming, Utter and Mhurchu, 2015, O’Loughlin et al. 2013, Gemming et al. 2013). However, issues with these methods can occur, such as procedures not being followed properly, poor image quality, challenges identifying composite dishes and users forgetting to capture images (Gemming, Utter and Mhurchu, Rollo et al. 2016). Some of the validated dietary recalls identified were web based which allows for more complete food databases to be included, supporting users to choose more specific food items. However, this should be achieved without increasing participant burden.

Using an appropriate method to validate a DAT is important (Livingstone, Robson and Wallace, 2004). Due to the difficulty of measuring absolute validity of dietary intake, studies typically measure relative validity, which includes errors associated with the reference method. Most of the tools identified had been tested for relative validity, as the most common reference method used was another self-reported DAT; this has limitations because it is susceptible to similar errors as the tool being validated. Ideally objective methods such as biomarkers should be used to validate DATs as they are not prone to the self-reporting or bias associated with other reference methods (Bingham, 2002, Hedrick et al. 2012). However, these methods only cover a limited number of dietary components and can be expensive and impractical when conducting a large study (Thompson et al. 2010, Hedrick et al. 2012, Freedman et al. 2014). In the present review only 17 tools were compared against biomarkers, some exclusively and some with additional reference methods. Additionally, the reference method should ideally take into account factors such as seasonality and variation between weekdays and weekends. Generally, this was seen when food diaries and dietary recalls were being validated but not FFQs.

The most common statistical method reported in the validation studies was the correlation coefficient. The use of correlation coefficient as the sole test has been criticised, since it only assesses whether an individual has preserved their ranking in relation to other participants and does not measure absolute agreement (Poslusna et al.2009, Bland and Altman, 1986). However, as FFQs are not necessarily measuring absolute intakes, others have stated this criticism does not apply (Masson et al. 2003). Lombard (2015) argues that a number of statistical approaches should be used in dietary validation studies, however, typically only one to three methods are used out of a possible six (correlation coefficient, paired t-test/Wilcoxon signed rank test, percent difference, cross-classification, weighted kappa, Bland-Altman LOA). Ideally validation studies should include LOA or intra class correlations (ICC) which measure agreement between a DAT and the reference method, as well as the extent of relative bias in the form of the MD (Bland and Altman, 1986). Given this, only results of validation studies that reported the LOA, or where this could be calculated in addition to the mean difference were included in our tabulated analysis. Similarly, comparing mean differences and LOAs in the summary plots are the focus on the [www.nutritools.org/](http://www.nutritools.org/) website to help researchers select DATs. Although researchers may be advised to select DATs with small mean differences and narrow LOAs (or at least avoid those with larger mean differences and wide LOAs), further guidance is needed on what may be classed as small/ narrow or large/wide, for instance expressed as a percentage of mean intakes of the population of interest, and/or as absolute values in units of the nutrient.

As observed from the range of the LOA, the estimated intakes can vary widely depending on the tool type and reference method used. The validation method can affect results for particular nutrients resulting in wider LOA. For example, assessing energy intake in children using a weighed food diary can be problematic due to reliance on proxy information from parents and / or carers (Lanigan et al. 2001). Limits of agreement were wide in a study validating a food diary against an FFQ (Broadfield et al. 2003), possibly partly due to limited frequency of consumption options and limited food lists in an FFQ tool. Accurate estimation of the Bland-Altman LOA between two methods can also be compromised by sample size. Studies with a sample size of ≥50 will enable greater accuracy of estimation for particular nutrients (Cade et al. 2002) with ≥100 subjects required to estimate true energy intakes to within 4% of a reference method (Day et al. 2001).

The variation and lack of statistical methods used in validation studies raises concerns about the quality of reporting in nutritional epidemiology. Missing and poor quality description of the validation methodology was found. Lack of information on the development of the DAT was common as a number of tools, especially those which had been adapted from previously developed tools, provided incorrect citations of the methodology papers, noted in other dietary assessment reviews (Bryant et al. 2014). The issues surrounding the variation and the quality of reporting can make recommending one DAT over another difficult (England et al. 2015). In order to improve the quality of reporting in nutritional epidemiology and dietary assessment research, new guidelines have been developed by the STROBE-nut consortium (Lachat et al. 2016). It is important that these guidelines are promoted, as a higher quality of reporting will allow for easier comparison and understanding of DATs. Additionally, validation study results are not necessarily representative of wider populations. For instance, some validations used or excluded specific populations which can hinder comparison and selection of DATs. Also volunteer sampling was the method used by the majority of validation studies through contact via GP surgery, school letters or posters and / or email advertisements.

Study strengths and limitations of study

The systematic and comprehensive approach adopted for this study was a strength as it was a practical way of obtaining information on DATs compared to undertaking multiple reviews of each type of DAT for different foods and / or nutrients which would have taken too long given available resources. Cross checking against DAT registers minimized the likelihood of missing tools. Another strength is the interactive nature of the website designed to search and display information about the DATs and their validations, which guides researchers to select appropriate DATs.

The main limitation of this study was that identification of all DATs validated in UK populations could not be guaranteed, as not all of them would have been included in a systematic or literature review. All of these tools are reported in detail on the Nutritools website plus detail on 66 international tools (not discussed in this paper). Also despite the date restriction on the published reviews (≥ January 2000) there was no date restriction on the actual DAT raising the question of whether tools developed over 25 – 30 years ago are still fit for purpose today. Additionally, the website will need maintaining to ensure it remains current, holding information on up-to-date tools, including those from other countries and cultures; however limited funds for this is available.

**Conclusions and recommendations**

This review identified 63 validated UK DATs which covered a wide range of life-stages and nutrients and collated information from these. The characteristics of these DATs, their validation studies and the validation results are now on the interactive [www.nutritools.org](http://www.nutritools.org) website. This can guide researchers to compare and choose the most suitable DAT for their research question, potentially leading to improvement of research in nutritional epidemiology.

This research provides knowledge to assist dietary assessment, having a positive impact on public health policy and society through the potential to support dietary advice and recommendations which can reduce the financial burden of non-communicable disease.

**Funding**

This work was supported by the UK Medical Research Council [Grant number MR/L02019X/1].

**Acknowledgments**

The members of the DIET@NET consortium* are: Dr Nisreen A. Alwan; Prof Janet E. Cade; Paul Finglas; Prof Tim Key; Prof Barrie Margetts; Dr Darren Greenwood; Prof Andy Ness; Prof Sian Robinson; Dr Toni Steer; Polly Page; Prof Petra A. Wark. The members of the DIET@NET project team have included: Prof Janet E. Cade; Dr Marisol Warthon-Medina; Katherine Greathead; Bethany Knowles; Neil Hancock; Victoria Burley; Jozef Hooson, Dr Jayne Hutchinson, Dr Elisa Vargas-Garcia, Linda A. Bush and Lauren E. Gibson.

The members of the Tool Selection Working Group were: Dr Victoria J. Burley; Dr Darren C. Greenwood; Prof Sian Robinson; Mark Roe; Dr Toni Steer and Prof Petra A. Wark. The members of the Access Working Group are: Prof Andy Ness; Polly Page; Paul Finglas and Prof Tim Key

**Author contributions**

JZH wrote the first draft of the manuscript with input from the DIET@NET experts. JZH and KG were responsible for screening to identify review eligibility. JZH and MWM updated the search. JYH updated the manuscript after peer review. All authors: JZH, MWM, NH, KG, BK, JYH, EVG, LEG, LAB, BM, SR, AN, NAA, PAA, MR, PF, TS, PP, LJ, KR, BA, VJB, DCG and JEC read and approved the final manuscript.

JZH and EVG processed the data, JZH and NH managed the data. JYH, LAB and LEG were involved in the analysis for table 4 with DCG providing advice on interpreting statistical results reported in the validations and reviews.  JZH, JYH, MWM LG and LAB checked data on the nutritools website.

SR led the tool selection working group. AN led the access working group. MWM was the DIET@NET project manager, succeeding KG. JEC was the principal investigator and supervised the DIET@NET project.

Conflict of Interest

The authors declare no competing financial interests. The University of Leeds has established a spin-out company, Dietary Assessment Ltd for myfood24, a new online dietary assessment tool; Professor Janet Cade is a director and shareholder of the company.

**References**

Agency FS (2013) Development of a web-based 24-hour dietary recall tool for use by 11–24 year olds: INTAKE24. *Newcastle University*

Albar SA, Alwan NA, Evans CE, Greenwood DC, Cade JE (2016) Agreement between an online dietary assessment tool (myfood24) and an interviewer-administered 24-h dietary recall in British adolescents aged 11–18 years. *British Journal of Nutrition* **115**: 1678-1686.

Arens-Volland AG, Spassova L, Bohn T (2015) Promising approaches of computer-supported dietary assessment and management—Current research status and available applications. *International Journal of Medical Informatics* **84:** 997-1008.

Ashfield-Watt P, Welch A, Godward S, Bingham S (2007) Effect of a pilot community intervention on fruit and vegetable intakes: use of FACET (Five-a-day Community Evaluation Tool). *Public Health Nutrition* **10**: 671-680.

Bach A, Serra-Majem L, Carrasco JL, Roman B, Ngo J, et al. (2006) The use of indexes evaluating the adherence to the Mediterranean diet in epidemiological studies: a review. *Public Health Nutrition* **9**: 132.

Bates, B., Lennox, A., Prentice, A., Bates, C. J., Page, P., Nicholson, S. & Swan, G. 2014. National Diet and Nutrition Survey: Results from Years 1, 2, 3 and 4 (combined) of the Rolling Programme (2008/2009-2011/2012): a Survey Carried Out on Behalf of Public Health England and the Food Standards Agency, *Public Health England*.

Bell LK, Golley RK, Magarey AA (2013) Short tools to assess young children's dietary intake: a systematic review focusing on application to dietary index research. *Journal of Obesity* **2013** 1-17.

Bingham S, Gill C, Welch A, Day K, Cassidy A, et al. (1994) Comparison of dietary assessment methods in nutritional epidemiology: weighed records v. 24 h recalls, food-frequency questionnaires and estimated-diet records. *British Journal of Nutrition* **72**: 619-643.

Bingham SA, Day NE (1997) Using biochemical markers to assess the validity of prospective dietary assessment methods and the effect of energy adjustment. *The American Journal of Clinical Nutrition* **65**: 1130S-1137S.

Bingham SA, Gill C, Welch A, Cassidy A, Runswick SA, et al. (1997) Validation of dietary assessment methods in the UK arm of EPIC using weighed records, and 24- hour urinary nitrogen and potassium and serum vitamin C and carotenoids as biomarkers. *International Journal of Epidemiology* **26**: S137.

Bingham SA (2002) Biomarkers in nutritional epidemiology. *Public Health Nutrition* **5:** 821-827.

Black AE, Welch AA, Bingham SA (2000) Validation of dietary intakes measured by diet history against24 h urinary nitrogen excretion and energy expenditure measuredby the doubly-labelled water method in middle-aged women. *British Journal of Nutrition* **83**: 341-354.

Bland, J.M. & Altman, D (1986). Statistical methods for assessing agreement between two methods of clinical measurement. *The Lancet* **327** (8476) 307-310

Bodner C, Soutar A, New S, Scaife A, Byres M, et al. (1998) Validation of a food frequency questionnaire for use in a Scottish population: correlation of antioxidant vitamin intakes with biochemical measures. *Journal of Human Nutrition and Dietetics* **11**: 373-380.

Bolton-Smith C, Casey C, Gey K, Smith W, Tunstall-Pedoe H (1991) Antioxidant vitamin intakes assessed using a food-frequency questionnaire: correlation with biochemical status in smokers and non-smokers. *British Journal of Nutrition* **65**: 337-346.

Bradley J, Simpson E, Poliakov I, Matthews JN, Olivier P, et al. (2016) Comparison of INTAKE24 (an online 24-h dietary recall tool) with interviewer-led 24-h recall in 11–24 year-old. *Nutrients* **8:** 358.

Broadfield E, McKeever T, Fogarty A, Britton J (2003) Measuring dietary fatty acid intake: validation of a food-frequency questionnaire against 7d weighed records. *British Journal of Nutrition* **90**: 215-220.

Brunner E, Juneja M, Marmot M (2001) Dietary assessment in Whitehall II: comparison of 7 d diet diary and food-frequency questionnaire and validity against biomarkers. *British Journal of Nutrition* **86**: 405-414.

Bryant M, Ashton L, Brown J, Jebb S, Wright J, et al. (2014) Systematic review to identify and appraise outcome measures used to evaluate childhood obesity treatment interventions (CoOR): evidence of purpose, application, validity, reliability and sensitivity. *Health Technology Assessment* **18** (51).

Burrows TL, Martin RJ, Collins CE (2010). A systematic review of the validity of dietary assessment methods in children when compared with the method of doubly labeled water. *Journal of the American Dietetic Association* **110:** 1501-1510.

Burrows T, Golley RK, Khambalia A, McNaughton SA, Magarey A, et al. (2012) The quality of dietary intake methodology and reporting in child and adolescent obesity intervention trials: a systematic review. *Obesity Reviews* **13**: 1125-1138.

Cade JE (2017) Measuring diet in the 21st century: use of new technologies. *Proceedings of the Nutrition Society* **76 (3**) 276-282.

Cade, J, Warthon-Medina, M, Albar, S, Alwan, N.A. & Ness, A et al (2017). DIET@NET: Best Practice Guidelines for dietary assessment in health research

Cade J, Burley V, Warm D, Thompson R, Margetts B (2004) Food-frequency questionnaires: a review of their design, validation and utilisation. *Nutrition Research Reviews* **17**: 5-22.

Cade J, Frear L, Greenwood D (2006) Assessment of diet in young children with an emphasis on fruit and vegetable intake: using CADET–Child and Diet Evaluation Tool. *Public Health Nutrition* **9:** 501-508.

Cade J, Thompson R, Burley V, Warm D (2002) Development, validation and utilisation of food-frequency questionnaires–a review. *Public Health Nutrition* 5: 567-587.

Calfas KJ, Zabinski MF, Rupp J (2000) Practical nutrition assessment in primary care settings: A review. *American Journal of Preventive Medicine* **18** (4): 289-299.

Carroll RJ, Midthune D, Subar AF, Shumakovich M, Freedman LS, et al. (2012) Taking advantage of the strengths of 2 different dietary assessment instruments to improve intake estimates for nutritional epidemiology. *American Journal of Epidemiology* 175 (4) 340-347.

Carvalho KMBd, Dutra ES, Pizato N, Gruezo ND, Ito MK (2014) Diet quality assessment indexes. *Revista de Nutrição* **27**: 605-617.

Carter MC, Albar SA, Morris MA, Mulla UZ, Hancock N, et al. (2015) Development of a UK online 24-h dietary assessment tool: Myfood24. *Nutrients* **7**: 4016-4032.

Carter MC, Burley V, Nykjaer C, Cade J (2013) ‘My Meal Mate’(MMM): validation of the diet measures captured on a smartphone application to facilitate weight loss. *British Journal of Nutrition* **109**: 539-546.

Christian MS, Evans CE, Nykjaer C, Hancock N, Cade JE (2015) Measuring diet in primary school children aged 8-11 years: validation of the Child and Diet Evaluation Tool (CADET) with an emphasis on fruit and vegetable intake. *European Journal of Clinical Nutrition* **69**: 234-241.

Cleghorn CL, Harrison RA, Ransley JK, Wilkinson S, Thomas J, et al. (2016) Can a dietary quality score derived from a short-form FFQ assess dietary quality in UK adult population surveys? *Public Health Nutrition*: **1** 1-9.

Comrie F, Masson LF, McNeill G (2009) A novel online Food Recall Checklist for use in an undergraduate student population: a comparison with diet diaries. Nutrition journal 8: 13.

Davies PS, Coward W, Gregory J, White A, Mills A (1994) Total energy expenditure and energy intake in the pre-school child: a comparison. *British Journal of Nutrition* **72**: 13-20.9696.

Day NE, McKeown N, Wong M-Y, Welch A, Bingham S (2001) Epidemiological assessment of diet: a comparison of a 7-day diary with a food frequency questionnaire using urinary markers of nitrogen, potassium and sodium. *International Journal of Epidemiology* **30**: 309-317.

de Lauzon-Guillain B, Oliveira A, Charles MA, Grammatikaki E, Jones L, et al. (2012) A review of methods to assess parental feeding practices and preschool children's eating behavior: the need for further development of tools. *Journal of the Academy of Nutrition and Dietetics* **112**: 1578-1602

Dong, J (2003). Dietary Targets Monitor. Available at: <https://www.gov.scot/topics>

Dunn S, Datta A, Kallis S, Law E, Myers CE, et al. (2011) Validation of a food frequency questionnaire to measure intakes of inulin and oligofructose. *European Journal of Clinical Nutrition* **65**: 402-408.

Edmunds L, Ziebland S (2002) Development and validation of the Day in the Life Questionnaire (DILQ) as a measure of fruit and vegetable questionnaire for 7–9 year olds. *Health Education Research* **17:** 211-220.

England C, Andrews R, Jago R, Thompson J (2015) A systematic review of brief dietary questionnaires suitable for clinical use in the prevention and management of obesity,

Falcão-Gomes RC, Coelho AAS, Schmitz BdAS (2006) Characterization of dietary intake assessment studies in pre-school children. *Revista de Nutrição* **19**: 713-727.

Fallaize R, Forster H, Macready AL, Walsh MC, Mathers JC, et al. (2014) Online dietary intake estimation: reproducibility and validity of the Food4Me food frequency questionnaire against a 4-day weighed food record. *Journal of Medical Internet Research* **16** (8) e190

Falomir Z, Arregui M, Madueño F, Corella D, Coltell Ó (2012) Automation of Food Questionnaires in Medical Studies: A state-of-the-art review and future prospects. *Computers in Biology and Medicine* **42:** 964-974.

Forster H, Fallaize R, Gallagher C, O’Donovan CB, Woolhead C, et al. (2014) Online dietary intake estimation: the Food4Me food frequency questionnaire. *Journal of Medical Internet Research* **16** (6) e150.

Foster E, Delve J, Simpson E, Breininger S-P (2014) Comparison study: INTAKE24 vs Interviewer led recall Final report. Citeseer

Frainer DES, Adami F, de Vasconcelos FdAG (2008) Systematic review about methods of energy expenditure and energy intake in children and adolescents. *Brazilian Journal of Kinanthropometry and Human Performance* **10:** 197-205.

Freedman LS, Commins JM, Moler JE, Arab L, Baer DJ, et al. (2014) Pooled results from 5 validation studies of dietary self-report instruments using recovery biomarkers for energy and protein intake. *American Journal of Epidemiolog*y: **180** (2) 172-188.

Gavrieli A, Naska A, Konstantinidi C, Berry R, Roe M, et al. (2014) Dietary monitoring tools for risk assessment. *EFSA Supporting Publications* 11.

Gemming L, Utter J, Mhurchu CN (2015) Image-assisted dietary assessment: a systematic review of the evidence. *Journal of the Academy of Nutrition and Dietetics* **115**: 64-77.

Gemming L, Doherty A, Kelly P, Utter J, Mhurchu CN (2013) Feasibility of a SenseCam-assisted 24-h recall to reduce under-reporting of energy intake. *European Journal of Clinical Nutrition* **67:** 1095-1099.

Hartwell DH, CJK (2001) Comparison of a self-administered quantitative food amount frequency questionnaire with 4-day estimated food records. *International Journal of Food Sciences and Nutrition* **52**: 151-159.

Heath A-LM, Roe MA, Oyston SL, Fairweather-Tait SJ (2005) Meal-based intake assessment tool: relative validity when determining dietary intake of Fe and Zn and selected absorption modifiers in UK men. *British Journal of Nutrition* **93**: 403-416.

Heald C, Bolton-Smith C, Ritchie M, Morton M, Alexander F (2006) Phyto-oestrogen intake in Scottish men: use of serum to validate a self-administered food-frequency questionnaire in older men. *European Journal of Clinical Nutrition* **60**: 129-135.

Hedrick VE, Dietrich AM, Estabrooks PA, Savla J, Serrano E, et al. (2012) Dietary biomarkers: advances, limitations and future directions. *Nutrition Journal* **11**: (1) 109.

Heller RF, Pedoe HDT, Rose G (1981) A simple method of assessing the effect of dietary advice to reduce plasma cholesterol. *Preventive Medicine* **10:** 364-370.

Henríquez-Sánchez P, Sánchez-Villegas A, Doreste-Alonso J, Ortiz-Andrellucchi A, Pfrimer K, et al. (2009) Dietary assessment methods for micronutrient intake: a systematic review on vitamins. *The British Journal of Nutrition* **102** (51): S10-S37.

Hillier FC, Batterham AM, Crooks S, Moore HJ, Summerbell CD (2012) The development and evaluation of a novel Internet-based computer program to assess previous-day dietary and physical activity behaviours in adults: the Synchronised Nutrition and Activity Program for Adults (SNAPA™). *British Journal of Nutrition* **107**: 1221-1231.

Hooper R, Heinrich J, Omenaas E, Sausenthaler S, Garcia-Larsen V, et al. (2010) Dietary patterns and risk of asthma: results from three countries in European Community Respiratory Health Survey-II. *British Journal of Nutrition* **103**: 1354-1365.

Holmes B, Dick K, Nelson M (2008) A comparison of four dietary assessment methods in materially deprived households in England. *Public Health Nutrition* **11**: 444-456.

Hollis JL, Craig LC, Whybrow S, Clark H, Kyle JA, et al. (2017) Assessing the relative validity of the Scottish Collaborative Group FFQ for measuring dietary intake in adults. *Public Health Nutrition* **20**: 449-455.

Hongu N, Hingle MD, Merchant NC, Orr BJ, Going SB, et al. (2011) Dietary assessment tools using mobile technology. *Topics in Clinical Nutrition* **26**: 300-311.

Illner A, Freisling H, Boeing H, Huybrechts I, Crispim S, et al. (2012) Review and evaluation of innovative technologies for measuring diet in nutritional epidemiology. *International Journal of Epidemiology* **41**: 1187-1203.

Jackson N, Little J, Wilson AD (1990) Comparison of diet history interview and self-completed questionnaire in assessment of diet in an elderly population. *Journal of Epidemiology and Community Health* **44**: 162-169.

Jia X, Craig LC, Aucott LS, Milne AC, McNeill G (2008) Repeatability and validity of a food frequency questionnaire in free-living older people in relation to cognitive function. *The Journal of Nutrition, Health & Aging* **12**: 735-741.

Johansson G (2008) Comparison of nutrient intake between different dietary assessment methods in elderly male volunteers. *Nutrition & Dietetics* **65**: 266-271.

Johnson B, Hackett A. (1997) Eating habits of 11–14‐year‐old schoolchildren living in less affluent areas of Liverpool, UK. *Journal of Human Nutrition and Dietetics* **10**: 135-144.

Johnson B, Hackett A, Roundfield M, Coufopoulos A (2001) An investigation of the validity and reliability of a food intake questionnaire. *Journal of Human Nutrition and Dietetics* **14**: 457-465.

Johnson RK (2002) Dietary intake—how do we measure what people are really eating? *Obesity Research* **10**: 63S-68S.

Johnson RK, Driscoll P, Goran MI (1996) Comparison of multiple-pass 24-hour recall estimates of energy intake with total energy expenditure determined by the doubly labeled water method in young children. *Journal of the American Dietetic Association* **96**: 1140-1144.

Kassam‐Khamis T, Nanchahal K, Mangtani P, Santos Silva Id, McMichael A, et al. (1999) Development of an interview‐administered food‐frequency questionnaire for use amongst women of South Asian ethnic origin in Britain. *Journal of Human Nutrition and Dietetics* **12**: 7-19.

Kim DJ, Holowaty EJ (2003) Brief, validated survey instruments for the measurement of fruit and vegetable intakes in adults: a review. *Preventive Medicine* **36:** 440-447.

Kolodziejczyk JK, Merchant G, Norman GJ (2012) Reliability and validity of child/adolescent food frequency questionnaires that assess foods and/or food groups. *Journal of Pediatric Gastroenterology and Nutrition* **55**: 4-13.

Kourlaba G, Panagiotakos DB (2009) Dietary quality indices and human health: A review. *Maturitas* **62** (109) 1-8.

Lachat C, Hawwash D, Ocké MC, Berg C, Forsum E, et al. (2016) Strengthening the Reporting of Observational Studies in Epidemiology–nutritional epidemiology (STROBE‐nut): An extension of the STROBE statement. *Nutrition Bulletin* **41:** 240-251

Lambert J, Agostoni C, Elmadfa I, Hulshof K, Krause E, et al. (2004) Dietary intake and nutritional status of children and adolescents in Europe. *British Journal of Nutrition* **92** (52): S147-S211.

Lanham SAB-S, C. (1993) Development of a food frequency questionaire. Proc Nutr Soc 52.

Lanigan J, Wells J, Lawson M, Lucas A (2001) Validation of food diary method for assessment of dietary energy and macronutrient intake in infants and children aged 6-24 months. *European Journal of Clinical Nutrition* **55**: 124.

Lean M, Anderson A, Morrison C, Currall J (2003) Evaluation of a dietary targets monitor. *European Journal of Clinical Nutrition* **57**: 667-673.

Lietz G, Barton KL, Longbottom PJ, Anderson AS (2002) Can the EPIC food-frequency questionnaire be used in adolescent populations? *Public Health Nutrition* **5**: 783-789.

Little P, Barnett J, Margetts B, Kinmonth A-L, Gabbay J, et al. (1999) The validity of dietary assessment in general practice. *Journal of Epidemiology and Community Health* **53**: 165-172.

Liu B, Young H, Crowe FL, Benson VS, Spencer EA, et al. (2011) Development and evaluation of the Oxford WebQ, a low-cost, web-based method for assessment of previous 24 h dietary intakes in large-scale prospective studies. *Public Health Nutrition* **14:** 1998-2005.

Livingstone MB, Prentice AM, Coward WA, Strain JJ, Black AE, et al. (1992) Validation of estimates of energy intake by weighed dietary record and diet history in children and adolescents. *The American Journal of Clinical Nutrition* **56**: 29-35.

Livingstone M, Robson P, Wallace J (2004) Issues in dietary intake assessment of children and adolescents. *British Journal of Nutrition* **92:** S213-S222.

Lombard MJ, Steyn NP, Charlton KE, Senekal M (2015) Application and interpretation of multiple statistical tests to evaluate validity of dietary intake assessment methods. *Nutrition Journal* **14:** 40.

Long JD, Littlefield LA, Estep G, Martin H, Rogers TJ, et al. (2010) Evidence review of technology and dietary *assessment*. *World Views on Evidence‐Based Nursing* **7**: 191-204.

McKeown NM, Day NE, Welch AA, Runswick SA, Luben RN, et al. (2001) Use of biological markers to validate self-reported dietary intake in a random sample of the European Prospective Investigation into Cancer United Kingdom Norfolk cohort. *The American Journal of Clinical Nutrition* **74**: 188-196.

McNaughton S, Mishra G, Bramwell G, Paul A, Wadsworth M (2005) Comparability of dietary patterns assessed by multiple dietary assessment methods: results from the 1946 British Birth Cohort. *European Journal of Clinical Nutrition* **59**: 341-352.

McPherson RS, Hoelscher DM, Alexander M, Scanlon KS, Serdula MK (2000) Dietary assessment methods among school-aged children: validity and reliability. *Preventive Medicine* 31: S11-S33.

Magarey A, Baulderstone L, Yaxley A, Markow K, Miller M (2015) Evaluation of tools used to measure calcium and/or dairy consumption in adults. *Public Health Nutrition* **18:** 1225-1236.

Margetts B, Cade J, Osmond C (1989) Comparison of a food frequency questionnaire with a diet record. *International Journal of Epidemiology* **18**: 868-873.

Marriott L, Robinson S, Poole J, Borland S, Godfrey K, et al. (2008) What do babies eat? Evaluation of a food frequency questionnaire to assess the diets of infants aged 6 months. *Public Health Nutrition* **11:** 751-756.

Marriott LD, Inskip HM, Borland SE, Godfrey KM, Law CM, et al. (2009) What do babies eat? Evaluation of a food frequency questionnaire to assess the diets of infants aged 12 months. *Public Health Nutrition* **12:** 967.

Marshall S, Burrows T, Collins C (2014) Systematic review of diet quality indices and their associations with health‐related outcomes in children and adolescents. *Journal of Human Nutrition and Dietetics* **27**: 577-598.

Masson L, McNeill G, Tomany J, Simpson J, Peace H, et al. (2003) Statistical approaches for assessing the relative validity of a food-frequency questionnaire: use of correlation coefficients and the kappa statistic. *Public Health Nutrition* **6**: 313-321.

Mohd-Shukri NA, Bolton JL, Norman JE, Walker BR, Reynolds RM (2013) Evaluation of an FFQ to assess total energy and nutrient intakes in severely obese pregnant women. *Public Health* *Nutrition* **16**: 1427-1435.

Molag ML, de Vries JH, Ocké MC, Dagnelie PC, van den Brandt PA, et al. (2007) Design characteristics of food frequency questionnaires in relation to their validity. *American Journal of Epidemiology* **166**: 1468-1478.

Montgomery C, Reilly JJ, Jackson DM, Kelly LA, Slater C, Paton JY, et al (2005). Validation of energy intake by 24-hour multiple pass recall: comparison with total energy expenditure in children aged 5–7 years. *British Journal of Nutrition* **93**(05):671-6.

Moore G, Tapper K, Murphy S, Clark R, Lynch R, et al. (2007) Validation of a self-completion measure of breakfast foods, snacks and fruits and vegetables consumed by 9-to 11-year-old schoolchildren. *European Journal of Clinical Nutrition* **61**: 420-430.

Moore HJ, Ells LJ, McLure SA, Crooks S, Cumbor D, et al. (2008) The development and evaluation of a novel computer program to assess previous-day dietary and physical activity behaviours in school children: The Synchronised Nutrition and Activity Program TM (SNAP TM). *British Journal of Nutrition* **99**: 1266-1274.

Moran VH (2007) A systematic review of dietary assessments of pregnant adolescents in industrialised countries. *British Journal of Nutrition* **97**: 411-425.

Mouratidou T, Mesana M, Manios Y, Koletzko B, Chinapaw M, et al. (2012) Assessment tools of energy balance‐related behaviours used in European obesity prevention strategies: review of studies during preschool. *Obesity Reviews* **13**: 42-55.

Mouratidou T, Ford F, Fraser RB (2006) Validation of a food-frequency questionnaire for use in pregnancy. *Public Health Nutrition* **9**: 515-522.

NCCOR.Available at: <https://tools.nccor.org/measures>

NCI NCI DIetary Assessment Calibration/Validation Register. Available at: - <https://epi.grants.cancer.gov/cgi-bin/dacv/index.pl?page=study_search>

Nelson M, Hague GF, Cooper C, Bunker VW (1988) Calcium intake in the elderly: validation of a dietary questionnaire. *Journal of Human Nutrition and Dietetic*s **1**: 115-127.

Nelson M, Nettleton P (1980) Dietary survey methods. 1. A semi-weighted technique for measuring dietary intake within families. *Journal of Human Nutrition* **34**: 325-348.

Ngo J, Engelen A, Molag M, Roesle J, García-Segovia P, et al. (2009) A review of the use of information and communication technologies for dietary assessment. *British Journal of Nutrition* **101**: S102-S112.

O'Donnell M, Nelson M, Wise P, Walker D (1991) A computerized diet questionnaire for use in diet health education. *British Journal of Nutrition* **66**: 3-15.

O'Loughlin G, Cullen SJ, McGoldrick A, O'Connor S, Blain R, et al. (2013) Using a wearable camera to increase the accuracy of dietary analysis. *American Journal of Preventive Medicine* **44**: 297-301.

Olukotun O, Seal N (2015) A Systematic Review of Dietary Assessment Tools for Children Age 11 Years and Younger. ICAN: *Infant, Child, & Adolescent Nutrition* **7**: 139-147.

Ortiz-Andrellucchi A, Henríquez-Sánchez P, Sánchez-Villegas A, Pena-Quintana L, Mendez M, et al. (2009) Dietary assessment methods for micronutrient intake in infants, children and adolescents: a systematic review. *British Journal of Nutrition* **102**: S87-S117.

Ortiz-Andrellucchi A, Sánchez-Villegas A, Doreste-Alonso J, de Vries J, de Groot L, et al. (2009) Dietary assessment methods for micronutrient intake in elderly people: a systematic review. *British Journal of Nutrition* **102**: S118-S149.

Ortiz-Andrellucchi A, Doreste-Alonso J, Henríquez-Sánchez P, Cetin I, Serra-Majem L (2009) Dietary assessment methods for micronutrient intake in pregnant women: a systematic review. *British Journal of Nutrition* **102**: S64-S86

Øverby NC, Serra-Majem L, Andersen LF (2009) Dietary assessment methods on n-3 fatty acid intake: a systematic review. *British Journal of nNutrition* **102**: S56-S63.

Palmer MA, Morgan CL (2012) How well are we validating food frequency questionnaires that measure dietary iron intakes of Australian and New Zealand adults? *Nutrition & Dietetics* **69:** 159-166

Papadaki A, Scott J (2007) Relative validity and utility of a short food frequency questionnaire assessing the intake of legumes in Scottish women. *Journal of Human Nutrition and Dietetics* **20**: 467-475.

Park JY, Vollset SE, Melse‐Boonstra A, Chajès V, Ueland PM, et al. (2013) Dietary intake and biological measurement of folate: a qualitative review of validation studies. *Molecular Nutrition & Food Research* **57**: 562-581.

Pedraza DF, Menezes TNd (2015) Food Frequency Questionnaire developed and validated for the Brazilian population: a review of the literature. *Ciência & Saúde Coletiva* **20**: 2697-2720

Poslusna K, Ruprich J, de Vries JHM, Jakubikova M, van't Veer P (2009) Misreporting of energy and micronutrient intake estimated by food records and 24 hour recalls, control and adjustment methods in practice. *British Journal of Nutrition* **101**: S73-S85

Probst YC, Tapsell LC (2005) Overview of computerized dietary assessment programs for research and practice in nutrition education. J*ournal of Nutrition Education and Behavior* **37**: 20-26

Publication N-Fa. Available at:  <https://epi.grants.cancer.gov/cgibin/dacv/index.pl?page=pub_search>

Pufulete M, Emery PW, Nelson M, Sanders TA (2002) Validation of a short food frequency questionnaire to assess folate intake. *British Journal of Nutrition* **87**: 383-390

Rankin D, Hanekom S, Wright H, MacIntyre U (2010) Dietary assessment methodology for adolescents: a review of reproducibility and validation studies. *South African Journal of Clinical Nutrition* **23**: 65-74

Reilly JJ, Montgomery C, Jackson D, MacRitchie J, Armstrong J (2001). Energy intake by multiple pass 24 h recall and total energy expenditure: a comparison in a representative sample of 3–4-year-olds. *British Journal of Nutrition* **86** (05):601-5

Riordan F, Ryan K, Perry IJ, Schulze MB, Andersen LF, et al. (2017) A systematic review of methods to assess intake of fruits and vegetables among healthy European adults and children: a DEDIPAC (DEterminants of DIet and Physical Activity) study. *Public Health Nutrition* **20:** 417-448.

Riordan F, Ryan K, Perry IJ, Schulze MB, Andersen LF, et al. (2017) A systematic review of methods to assess intake of sugar-sweetened beverages among healthy European adults and children: a DEDIPAC (DEterminants of DIet and Physical Activity) study. *Public Health Nutrition* **20**: 578-597.

Roberts K, Flaherty S (2010) Review of dietary assessment methods in public health. *National Obesity Observatory:* Oxford

Robinson S, Marriott L, Poole J, Crozier S, Borland S, et al. (2007) Dietary patterns in infancy: the importance of maternal and family influences on feeding practice. *British Journal of Nutrition* **98:** 1029-1037.

Roddam AW, Spencer E, Banks E, Beral V, Reeves G, et al. (2005) Reproducibility of a short semi-quantitative food group questionnaire and its performance in estimating nutrient intake compared with a 7-day diet diary in the Million Women Study. *Public Health Nutrition* **8**: 201-213.

Rodrigues AGM, Proença RPdC (2011) Use of food images for evaluating food intake. *Revista de* *Nutrição* **24**: 765-776

Roe L, Strong C, Whiteside C, N A, Mant D (1994) Dietary intervention in primary care: validity of the DINE method for diet assessment. *Family Practice* **11**: 375-381.

Rollo ME, Williams RL, Burrows T, Kirkpatrick SI, Bucher T, et al. (2016) What Are They Really Eating? A Review on New Approaches to Dietary Intake Assessment and Validation. *Current* *Nutrition Report*s **5**: 307-314.

Román-Vinas B, Barba LR, Ngo J, Martínez-González MÁ, Wijnhoven TM, et al. (2009) Validity of dietary patterns to assess nutrient intake adequacy. *British Journal of Nutrition* **101**: S12-S20.

Roman‐Viñas B, Ortiz‐Andrellucchi A, Mendez M, Sánchez‐Villegas A, Quintana LP, et al. (2010) Is the food frequency questionnaire suitable to assess micronutrient intake adequacy for infants, children and adolescents? *Maternal & Child Nutrition* **6:** 112-121.

Samaras K, Kelly PJ, Chiano MN, Arden N, Spector TD, et al. (1998) Genes versus environment: the relationship between dietary fat and total and central abdominal fat. *Diabetes Care* **21**: 2069-2076

Sasaki S, Kim MK (2003) Validation of self-administered dietary assessment questionnaires developed for Japanese subjects: systematic review. *J Community Nutr* **5**: 83-92.

Satija A, Yu E, Willett WC, Hu FB (2015) Understanding nutritional epidemiology and its role in policy. Advances in Nutrition: *An International Review Journal* **6**: 5-18.

Serdula MK, Alexander MP, Scanlon KS, Bowman BA (2001) What Are Preschool Children Eating? A Review of Dietary Assessment 1. *Annual Review of Nutrition* **21**: 475-498.

Serra-Majem L, Nissensohn M, Øverby NC, Fekete K (2012) Dietary methods and biomarkers of omega 3 fatty acids: a systematic review. *British Journal of Nutrition* **107:** S64-S76.

Serra-Majem L, Pfrimer K, Doreste-Alonso J, Ribas-Barba L, Sánchez-Villegas A, et al. (2009) Dietary assessment methods for intakes of iron, calcium, selenium, zinc and iodine. *British Journal of Nutrition* **102**: S38-S55.

Sevak L, Mangtani P, McCormack V, Bhakta D, Kassam-Khamis T, et al. (2004) Validation of a food frequency questionnaire to assess macro-and micro-nutrient intake among South Asians in the United Kingdom. *European Journal of Nutrition* **43**: 160-168.

Sharp DB, Allman-Farinelli M (2014) Feasibility and validity of mobile phones to assess dietary intake. Nutrition 30: 1257-1266.

Shim J-S, Oh K, Kim HC (2014) Dietary assessment methods in epidemiologic studies. *Epidemiology* *and Health* **36**: e2014009.

Shriver BJ, Roman-Shriver CR, Long JD (2010) Technology-based methods of dietary assessment: recent developments and considerations for clinical practice. *Current Opinion in Clinical Nutrition & Metabolic Care* **13**: 548-551.

Silva TdA, Vasconcelos SML (2012) Methodological procedures used in food frequency questionnaires made in Brazil: a systematic review. *Revista de Nutrição* **25:** 785-797.

Sofianou-Katsoulis A, Mesher D, Sasieni P, Du Toit G, Fox AT, et al. (2011) Assessing peanut consumption in a population of mothers and their children in the uk: validation study of a food frequency questionnaire. *World Allergy Organization Journal* **4**: 38.

Statacorp (2015). Stata statistical software: Release 14. College Sttion TX: Statacorp LP

Sutton E, Emmett P, Lawlor DA (2008) Measuring dietary sodium intake in infancy: A review of available methods. *Paediatric and Perinatal Epidemiology* **22:** 261-268.

Tabacchi G, Amodio E, Di Pasquale M, Bianco A, Jemni M, et al. (2014) Validation and reproducibility of dietary assessment methods in adolescents: a systematic literature review. *Public Health Nutrition* **17**: 2700-2714.

Tabacchi G, Filippi AR, Amodio E, Jemni M, Bianco A, et al. (2016) A meta-analysis of the validity of FFQ targeted to adolescents. *Public Health Nutrition* **19:** 1168-1183.

Thompson FE, Subar AF (2008) Dietary assessment methodology. *Nutrition in the Prevention and Treatment of Disease* **2**: 3-39.

Thompson FE, Subar AF, Loria CM, Reedy JL, Baranowski T (2010) Need for technological innovation in dietary assessment. *Journal of the American Dietetic Association* **110:** 48

Thompson RL, Margetts BM, (1993) Comparison of a food frequency questionnaire with a 10-day weighed record in cigarette smokers. *International Journal of Epidemiology* **22**: 824-833.

Timon CM, van den Barg R, Blain RJ, Kehoe L, Evans K, et al. (2016) A review of the design and validation of web-and computer-based 24-h dietary recall tools. *Nutrition Research Reviews* **29**: 268-280.

Timon CM, Astell AJ, Hwang F, Adlam TD, Smith T, et al. (2015) The validation of a computer-based food record for older adults: the Novel Assessment of Nutrition and Ageing (NANA) method. *British Journal of Nutrition* **113**: 654-664.

Timmins KA, Vowden K, Husein F, Burley V (2014) Making the best use of new technologies in the National Diet and Nutrition Survey: a review

Trabulsi J, Schoeller DA (2001) Evaluation of dietary assessment instruments against doubly labeled water, a biomarker of habitual energy intake. *American Journal of Physiology-Endocrinology And Metabolism* **281:** E891-E899.

Venter C, Higgins B, Grundy J, Clayton C, Gant C, et al. (2006) Reliability and validity of a maternal food frequency questionnaire designed to estimate consumption of common food allergens. *Journal of Human Nutrition and Dietetics* **19**: 129-138.

Verkasalo PK, Appleby PN, Allen NE, Davey G, Adlercreutz H, et al. (2001) Soya intake and plasma concentrations of daidzein and genistein: validity of dietary assessment among eighty British women (Oxford arm of the European Prospective Investigation into Cancer and Nutrition). *British Journal of Nutrition* **86**: 415-421.

Vézina-Im L-A, Robitaille J (2014) Validity and reliability of self-reported measures of foods and nutrients in pregnancy: a systematic review. *Current Nutrition Reports* **3:** 245-280.

Vucic V, Glibetic M, Novakovic R, Ngo J, Ristic-Medic D, et al. (2009) Dietary assessment methods used for low-income populations in food consumption surveys: a literature review. *British Journal of Nutrition* **101**: S95-S101.

Wakai K (2009) A review of food frequency questionnaires developed and validated in Japan. *Journal of Epidemiology* **19**: 1-11.

Walker JL, Bell KL, Caristo FM, Boyd RN, Davies PS (2011) A review of energy intake measures used in young children with cerebral palsy. *Developmental Medicine & Child Neurology* **53:** 569-569.

Willett W (2001) Commentary: dietary diaries versus food frequency questionnaires—a case of undigestible data. *International Journal of Epidemiology* **30**: 317-319.

Wirt A, Collins CE (2009) Diet quality–what is it and does it matter? *Public Health Nutrition* **12:** 2473-2492.

Wojtusiak J, Gewa C, Pawloski L (2011) Dietary assessment in Africa: Integration with innovative technology. African Journal of Food, Agriculture, Nutrition and Development 11: 5629-5645

Wrieden W, Peace H, Armstrong J, Barton K (2003). A short review of dietary assessment methods used in National and Scottish Research Studies.

Yarnell J, Fehily A, Milbank J, Sweetnam P, Walker C (1983) A short dietary questionnaire for use in an epidemiological survey: comparison with weighed dietary records. *Human Nutrition Applied Nutrition* **37**: 103-112.

Yang WY, Burrows T, MacDonald‐Wicks L, Williams LT, Collins C, et al. (2014) Quality of dietary assessment methodology and reporting in epidemiology studies examining relationship between dietary outcome and childhood obesity in developing Asian countries: A systematic review. *Nutrition & Dietetics* **71:** 201-209.

Yaroch AL, Resnicow K, Khan LK (2000) Validity and reliability of qualitative dietary fat index questionnaires: a review. *Journal of the American Dietetic Association* **100:** 240-244.

Records identified through database search (n = 8413)

Additional records identified through other sources (n =7)

Records for screening after duplicates removed (n = 4433)

Records excluded after screening (n = 4297)

Records remaining and assessed for eligibility (n=136)

Reviews excluded with reasons (n = 68)

Not a review (n = 31)

Not reviewing dietary assessment tools (n = 14)

Article not found (n = 13)

Abstract paper (n = 5)

Reviewing screeners for malnutrition (n = 3)

Reviewing only image assisted methods (n = 1)

Reviewing only Personal Digital Assistant (n = 1)

Reviews assessed for eligibility (n = 68). Systematic reviews (n=29), non-systematic reviews (n=39)

Articles extracted and screened from the 68 reviews (n=2972)

Full text articles excluded with reasons (n = 99)

Paper not assessing dietary assessment tool or validation (n = 56)

Tool does not validate dietary intake (n = 19)

Paper unavailable (n=10)

Dietary assessment tool not validated (n = 10)

Abstract (n = 3)

Reliability paper (n=1)

Articles remaining that included a relevant UK DAT (n = 169)

Articles remaining after exclusion criteria (n = 70)

DATs identified from articles (51)

Additional DATs from cross-checking and internet searches (n=12)

DATS validated only on food groups (n=10: 5 adults only; 1 adults and children; 4 children only)

Note: adults = adults and/or elderly; children = infants, children and or adolescents

Total validated DATs identified

(n=63: 44 adults only; 6 adults and children; 13 children only)

DATs with energy/ macro/micronutrient intake validations (n=53: 39 adults only; 5 adults and children; 9 children only)

Figure 1. PRISMA flow chart indicating number of articles included at each phase.


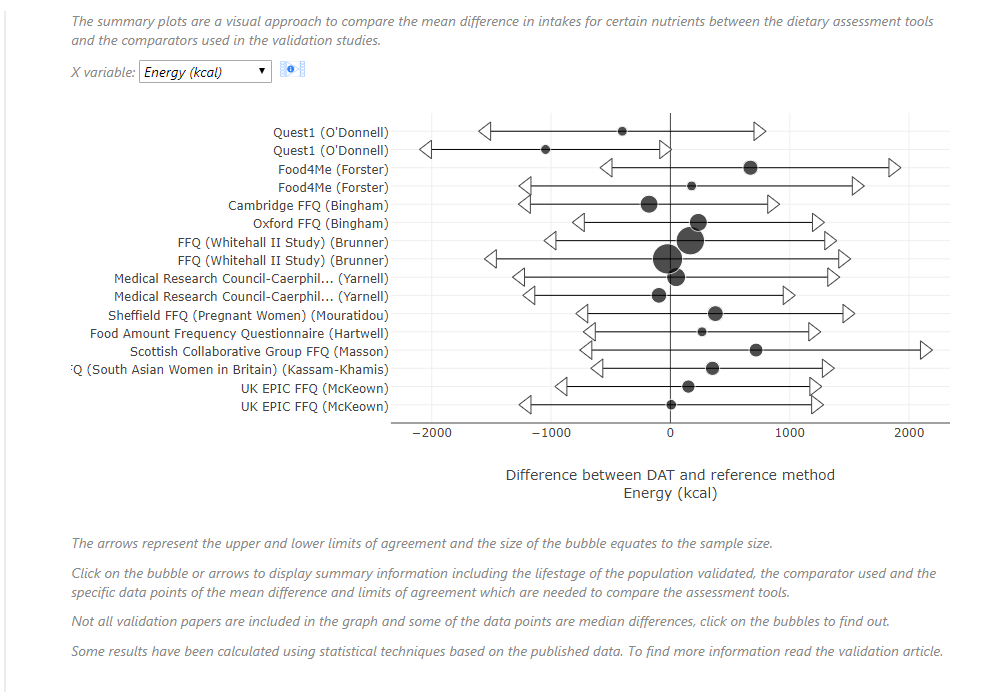


Figure 2 An example of a summary plot on the www.nutritools.org website

# Tables.

Table 1. Inclusion and exclusion criteria applied to the reviews and DATs

| **Reviews** | | **DATs** | |
| --- | --- | --- | --- |
| Inclusion criteria | Exclusion criteria | Inclusion criteria | Exclusion criteria |
| - Reviews that validated a DAT against a biomarker or another self-reported tool against energy, macro or micro nutrients or food groups - Reviews published since 1^st^ January 2000 | - Reviews that exclusively evaluated tools assessing inadequacy of diets in terms of malnutrition - Commentaries, editorials or other opinion articles | - Tools validated in a UK population   Be able to measure dietary intake   - Validation results can be entered on the nutritools website | - DATs measuring eating disorders, food preferences, feeding practices or inadequacy of diets - Lifestyle based tools (e.g. diet plus physical activity) - DATS measuring the purchasing of foods / drinks - Tools that assessed specific dietary interventions (e.g. Atkins, Mediterranean diet) - Non-UK tools |

Table 2 Number and description of dietary assessment tools for each life stage

| **Validation life stage and number of tools** | **Description** |
| --- | --- |
| Infants (≤3 yrs old) *(n=4)* | 2 FFQ and 2 food diaries |
| Children (3-11 yrs old) *(n=12)* | 5 recalls, 3 diaries, 2 checklists, 1 FFQ and 1 diet history |
| Adolescents (12-18 yrs old) *(n=10)* | 4 recalls, 2 food diaries, 2 food checklists, 1 FFQ and 1 diet history |
| *Pregnant women *(n=3)* | All FFQ’s |
| Adults (age 19-64) *(n=47)* | 30 FFQ’s, 8 24-hour recalls, 6 food diaries, 2 food checklists and 1 diet history |
| Elderly (>65) *(n=19)* | 9 FFQ’s, 4 food diaries, 2 food checklists, 3 recalls and 1 diet history |

* Also included in the adult cohort numbers

Only 2 of the validation studies exclusively included participants >65

Only 5 of the tools validated in children covered the full age range of 3-11 years old

2 of the infant validated tools measured dietary intake for a specific infant age = 6 months and 12 months

**Table 3**. General characteristics of the 63 UK dietary assessment tools and their validation studies

| **Dietary Assessment Tool Validation Studies** | | | | | | | | | |
| --- | --- | --- | --- | --- | --- | --- | --- | --- | --- |
| **First author (year)** | **Administration method / length of questionnaire** | **Nutrient database** | **First author and year** | **Food & nutrients (number of nutrients validated)** | **Life stage, age (mean /range) and *sample size (M/F)*** | **Reference method** | **Time span** | | **Statistical Method Used** |
|  |  |  |  |  |  |  | **DAT** | **Reference Method** |  |

**Weighed Food Diary**

| Bingham  (1994) | Self | MCW4 | Bingham  (1997) | Urinary nitrogen  Micronutrients (2) | Adults (50–65 yr)  *156 (0/56)* | Biomarkers | 16d | 8d over 12 months | Individual Means; Correlation Coefficient (S); Cross Classification |
| --- | --- | --- | --- | --- | --- | --- | --- | --- | --- |
| Davies  (1994) | By-Proxy | MCW4 | Davies  (1994) | Energy | Children & Infants (1.5 – 4.5 yr)  *81 (42/39)* | DLW | 4d consecutive | 10d | Mean Difference ; Correlation Coefficient; Limits of Agreement |
| Livingstone  (1992) | Self; By-Proxy | MCW4 inc. supplementary food composition data | Livingstone  (1992) | Energy | Children & Adolescents (7-18 yr)  *58 (29/29)* | DLW | 7d consecutive | 10 – 14d | Mean Difference(%); Limits of Agreement |

**Estimated Food Diary**

| Bingham  (1994) | Self | MCW4 | Bingham  (1994) | Energy; Macronutrients (7); Micronutrients (6) | Adults (50-65 yr)  *81 (0/81)* | Weighed Food Diary | 7d | 4 x 4d over 12 months | Individual Means; Correlation Coefficient (S); Cross Classification |
| --- | --- | --- | --- | --- | --- | --- | --- | --- | --- |
|  |  |  | Bingham  (1997) | Urinary nitrogen  Micronutrients (3) | Adults (50-65 yr)  *80 (0/80*) | Biomarkers | 7d | 8d over 12 months | Correlation Coefficient (P) |
|  |  |  | Johansson  (2008) | Energy; Macronutrients (6); Micronutrients (6); Food Groups | Elderly (65-88 yr)  *80 (80/0)* | Weighed Food Diary | 7d | 4 x 4d over 12 months | Individual Means |
| Carter (mymealmate)  (2013) | **Self | The Weight Loss Resources | Carter  (2013) | Energy; Macronutrients (3) | Adults (mean 35yr)  *50 (14/36*) | 24-Hour Recall | 7d consecutive | 2d | Mean Difference; Correlation Coefficient (P); Limits of Agreement |
| McKeown  (2001) | Self | DINER | McKeown  (2001) | Urinary nitrogen  Micronutrients (3) | Adults & Elderly (45-74 yr)  *146 (58/88)* | Biomarkers | 7d | 3d | Individual Means; Correlation Coefficient (P & S); Cross Classification |
|  |  |  | Day | Micronutrient (2) | Adults (45-74yr)  123 | Biomarkers | 7d | 6d over 12 months | Individual Means; Correlation Coefficient |
| Lanigan  (2001) | By-Proxy | COMP-EAT v.5 | Lanigan  (2001) | Energy; Macronutrient (3) | Infants (6-24 months)  *DLW = 21*  *Weighed Food Diary =72* | DLW & Weighed Food Diary | 5d | 7d (DLW) & 5d (Food Diary) | Mean Difference (%); Limits of Agreement |
| Timon  (NANA method)  (2015) | **Self | WinDiets | Timon  (2015) | Energy, Macronutrients (5); Micronutrients (10);  Food Group | Elderly (65-89 yr)  *94 (34/60)* | Estimated Food Diary & Biomarkers | 4d | 4d (Food Diary) & 1d (Biomarkers) | Mean Difference; Correlation Coefficient (P & S); Limits of Agreement |

**Semi-Weighed Food Diary**

| Holmes  (2008) | Self; By-Proxy; Interview | MCW5 | Holmes  (2008) | Energy; Macronutrients (4); Micronutrients (6);  Food Group | Children, Adolescents, Adults, Elderly (2-90 yr)  *44, 30, 111, 34*  Low SES | Weighed Food Diary | 4d | 4d | Mean Difference. |
| --- | --- | --- | --- | --- | --- | --- | --- | --- | --- |

**24-hour recall**

| *Bingham  (1994) | Self | MCW4 | Bingham (Structured & Unstructured)  (1994) | Energy, Macronutrients (7); Micronutrients (6) | Adults (50-65 yr)  *160 (0/160)* | Weighed Food Diary | 1d | 4 x 4d over 12 months | Individual Means; Correlation Coefficient (S); Cross Classification |
| --- | --- | --- | --- | --- | --- | --- | --- | --- | --- |
|  |  |  | Bingham (Structured & Unstructured)  (1997) | Urinary nitrogen  Micronutrients (3) | Adults (50-65 yr)  *156 (0/156)* | Biomarkers | 1d | 8d over 12 months | Correlation Coefficient (P & S) |
|  |  |  | Johansson (Unstructured)  (2008) | Energy; Macronutrients (6); Micronutrients (6); Food Groups | Elderly (65-88 yr)  *80 (80/0)* | Weighed Food Diary | 7d | 4 x 4d over 12 months | Individual Means |
| Carter (myfood24)  (2015) | **Self; Interview | MCW7 | Albar  (2016) | Energy; Macronutrients (6); Micronutrients (1); Food Groups | Adolescents  *75 (47/38)* | Multiple-Pass 24-Hour Recall | 2d (non-consecutive) | 2d (non-consecutive) | Mean Difference; Correlation Coefficient (ICC); Class Classification Limits of Agreement; Weighted Cohen’s kappa |
| *Comrie (FoRC)  (2009) | **Self | MCW6 | Comrie  (2009) | Energy; Macronutrients (2);  Food Groups | Adults (18-49 yr)  *53 (12/41)* | Estimated Food Diary | 4d | 4d | Mean Difference; Correlation Coefficient (S); Limits of Agreement |
| Edmunds (DILQ)  (2002) | Self | Not Reported | Edmunds  (2002) | Food Groups | Children (7-9 yr)  *204* | Direct Observation | 1d | 1d | Individual Means (count); Cross Classification (% matched); Cohen’s kappa |
| *Foster _[_(INTAKE24)  (2013) | **Self | MCW | Bradley  (2016) | Energy;  Macronutrients (6); Micronutrients (3); Food Groups | Adolescents & Adults (11-24 yr)  *168 (74/94)* | 24-Hour Recall | 4d (Results reported data on participants completing any number of days) | 4d (Results reported data on participants completing any number of days) | Mean ratios; Limits of Agreement |
| Hillier  (SNAPA)  (2012) | **Self | MCW6 | Hillier  (2012) | Food Groups | Adults (mean 34)  *44 (16/28)* | Direct Observation | 5d | 4d | Mean Difference; Cross Classification |
| *Holmes  (2008) | By-Proxy; Interview | MCW5 | Holmes  (2008) | Energy; Macronutrients (4); Micronutrients (6);  Food Group | Children, Adolescents, Adults, Elderly (2-90 yr)  *76, 48, 206, 54*  Low SES | Weighed Food Diary | 4d | 4d | Mean Difference. |
| *Johnson  (1996) | Interview | Food Intake Analysis | Reilly  (2001) | Energy | Children (3 – 4 yr)  *41 (23/18)* | DLW | 3d | 7d | Mean Difference; Limits of Agreement |
|  |  |  | Montgomery  (2005) | Energy | Children (4.5–7 yr)  *63 (32/31)* | DLW | 3d (Inc. 1 weekend d) | 2d | Mean Difference (bias); Limits of Agreement |
| Little  (1999) | Interview | Not Reported | Little  (1999) | Macronutrients (1); Micronutrients (1);  Food Groups | Adults & Elderly (18-80 yr)  *111 (53/58)* | Weighed Food Diary | 1d | 7d | Median Difference (%) Correlation Coefficient (S) |
| Liu  (Oxford WebQ)  (2011) | **Self | MCW5 | Liu  (2011) | Energy; Macronutrients (9); Micronutrients (10);  Food Group | Adults (19-82 yr)  *116 (32/84)* | Multiple-Pass 24-Hour Recall | 1d | 1d | Mean Difference (%); Correlation Coefficient (S); Cross Classification |
| Moore  (SNAP)  (2008) | **Self | Not Reported | Moore  (2008) | Food Groups | Children & Adolescents (7-15 yr)  *121 (49/72)* | Multiple Pass 24-Hour Recall | 1d | 1d | Individual Means (Count); Cross Classification |
| Moore  (Dietary Recall Questionnaire)  (2007) | **Self | Not Reported | Moore  (2007) | Food Groups | Children (9-11 yr)  *374 (157/ 215)*  Low SES | Multiple Pass 24-Hour Recall | 1d & an extra morning | 1d & an extra morning | Correlation Coefficient (S); Cross Classification; Cohen’s kappa |

**48-Hour Recall**

| McNaughton  (2005) | Interview | MCW | McNaughton  (2005) | Energy, Macronutrients (4); Micronutrients (9)  Food Group | Adults (43 yr)  *2265 (1116/ 1149)* | Estimated Food Diary | 2d | 5d | Mean Difference; Correlation Coefficient (S) |
| --- | --- | --- | --- | --- | --- | --- | --- | --- | --- |

**Food Frequency Questionnaire**

| Ashfield-Watt  (FACET)  (2007) | Self  ≤50 food items / questions | N/A | Ashfield-Watt  (2007) | Food Groups | Adults (age not reported)  *269*  Low SES | Estimated Food Diary | 1d | 1d | Individual Means; Correlation Coefficient; Cross Classification |
| --- | --- | --- | --- | --- | --- | --- | --- | --- | --- |
| Bingham (Cambridge FFQ)  (1994) | Self  ≥100 food items / questions | MCW4 | Bingham  (1994) | Energy; Macronutrients (7); Micronutrients (6)  Food Groups | Adults (50- 65 yr)  *160 (0/160)* | Weighed Food Diary | 1d | 4 x 4d over 12 months | Individual Means; Correlation Coefficient (S); Cross Classification |
| Bingham (Oxford FFQ)  (1994) | Self  ≥100 food items / questions | MCW4 | Bingham  (1994) | Energy;  Macronutrients (7); Micronutrients (6) | Adults (50- 65 yr)  *160 (0/160)* | Weighed food diary | 1d | 4 x 4d over 12 months | Individual Means; Correlation Coefficient (S); Cross Classification |
|  |  |  | Bingham  (1997) | Micronutrients (3) | Adults (50- 65 yr)  *160 (0/160)* | Biomarkers | 1d | 8d over 12 months | Correlation Coefficient (P & S) |
|  |  |  | Johansson  (2008) | Energy; Macronutrients (6); Micronutrients (6); Food Groups | Elderly (65-88 yr)  *80 (80/0)* | Weighed food diary | 1d | 4 x 4d over 12 months | Individual Means |
|  |  |  | Samaras  (1998) | Energy;  Macronutrients (4); | Adults (mean 58yr)  *162 (0/162)* | Estimated food diary | 1d | 7d | Individual Means; Correlation Coefficient |
|  |  |  | Verkasalo  (2001) | Food Groups | Adults (20-39 yr)  *80 (0/80)* | Biomarkers | 1d | 1d | Correlation Coefficient (S) |
|  |  |  | Little  (1999) | Macronutrients (1); Micronutrients (1);  Food Groups | Adults & Elderly (18-80 yr)  *111 (53/58)* | Weighed Food Diary | 1d | 7d | Median Difference (%) Correlation Coefficient (S); |
| Broadfield (DIETQ)  (2003) | Self  ≥100 food items / questions | DIETQ | Broadfield  (2003) | Macronutrients (5)  Food groups | Adults (mean 42yr)  *31 (15/16)* | Weighed Food Diary | 1d | 7d | Mean Difference; Correlation Coefficient (P+ S); Limits of Agreement |
| Brunner  (2001) | Self  ≥100 food items / questions | MCW4 &  MCW5 | Brunner  (2001) | Energy  Macronutrients (9); Micronutrients (8)  Food Group | Adults  (39-61yr)  860  *(457/403)* | Estimated Food Diary | 1d | 7d | Individual Means; Correlation Coefficient (S); Cross Classification |
| Cleghorn  (2016) | Self  ≤50 food items / questions | DANTE | Cleghorn  (2016) | Macronutrients (1);  Food Groups | Adults  FFQ- *705 (314/ 391*); 24hr Recall - *47 (25/22)* | FFQ & 24hr Recall | 1d | 1d | Mean Difference; Correlation Coefficient (S); Cohen’s kappa |
| Dunn^[30]^  (2010) | Self  ≤50 food items / questions | Not reported | Dunn  (2010) | Macronutrients (2)  Food Groups | Adults (18-50 yr)  *66 (17/49)* | Weighed Food Diary | 7d | 7d | Mean Difference; Cross Classification; Limits of Agreement |
| Forster  (Food4Me)  (2014) | Self  ≥100 food items / questions | National Adult Nutrition Survey (NANS) | Forster  (2014) | Energy;  Macronutrient (7);  Micronutrients (14);  Food Groups | Adults (30yr)  *113 (46/67)* | FFQ | 1d | 1d | Mean Difference; Correlation Coefficient (S); Class Classification; Limits of Agreement |
|  |  |  | Fallaize  (2014) | Energy; Macronutrients (7); Micronutrients (13); Food Groups | Adults (mean 27yr)  *49 (15/34)* | Weighed Food Diary | 1d | 4d | Mean Difference; Correlation Coefficient (S); Class Classification; Limits of Agreement |
| Hartwell  (2001) | Self  ≥100 food items / questions | DIET5 | Hartwell  (2001) | Energy;  Macronutrients (8); Micronutrients (4)  Food Group | Adults (mean 45-75yr)  *25 (16/9)* | Estimated Food Diary | 2d | 8d | Mean Difference; Correlation Coefficient (P); |
| Heath (MBIAT)  (2005) | Interview  ≥100 food items / questions | MCW4 &  MCW5 | Heath  (2005) | Micronutrients (4)  Food Groups | Adults (46-75 yr)  *48 (48/0)* | Weighed Food Diary | 3d | 12d | Mean Difference; Correlation Coefficient (S); Cross Classification |
| Heller  (1981) | Self  ≤50 food items / questions | Not Reported | Heller  (1981) | Macronutrients (1) | Adults (40-59 yr)  *68 (68/0)* | Weighed Food Diary | 1d | 3d | Correlation Coefficient |
| Hooper  (2010) | Self  ≥100 food items / questions | MCW6 | Hooper  (2010) | Energy; Macronutrients (3);  Food Groups | Adults (mean 29-55yr)  *263* | 24-hour recall | 1d | 1d | Correlation Coefficient (P) |
| Kassam-Khamis  (1999) | Interview  >51 to 99 food items / questions | COMP-EAT4; data on traditional South Asian foods & MCW5 | Kassam-Khamis (1999) | Energy; Macronutrients (4) | Adults (25-50 yr)  *11 (0/11)* | Weighed Food Diary | 1d | 7d | Median Paired Difference; Correlation Coefficient (P); Cross Classification |
|  |  |  | Sevak  (2004) | Energy; Macronutrients (8); Micronutrients (7) | Adults (34-75 yr)  *11 (0/11)* | 24-Hour Recall | 1d | 12 x 1d over 12 months | Individual Means; Correlation Coefficient (P & S); Cross Classification; Cohen’s kappa |
| Lanham  (1993) | Self  >51 to 99 food items / questions | MCW | Bodner  (1998) | Micronutrients (4) | Adults (39-45 yr)  *273 (118/ 155)* | Biomarkers | 1d | 1d | Individual Means; Correlation Coefficient (P); Cross Classification |
| Dong (2003) | Self  ≤50 food items / questions | Not Reported | Lean  (2003) | Food Groups | Adults (25-64 yr)  *1085 (522/ 563)* | FFQ | 1d | 1d | Median Difference (%); Correlation Coefficient |
| Little  (HEA1  (1999) | Self; Interview  >51 to 99 food items / questions | Royal Society of Cambridge Database | Little  (1999) | Micronutrients (1);  Food Groups | Adults & Elderly (18-80 yr)  *111 (53/58)* | Weighed Food Diary | 1d | 7d | Median Difference (%); Correlation Coefficient (S) |
| Little  (HEA2)  (1999) | Self; Interview  >51 to 99 food items / questions | Royal Society of Cambridge Database | Little  (1999) | Micronutrients (1); Food Groups | Adults & Elderly (18-80 yr)  *111 (53/58)* | Weighed Food Diary | 7d | 7d | Median Difference (%); Correlation Coefficient (S) |
| Little  (HEA3)  (1999) | Self; Interview  >51 to 99 food items / questions | Royal Society of Cambridge Database | Little  (1999) | Micronutrients (1); Food Groups | Adults & Elderly (18-80 yr)  *111 (53/58)* | Weighed Food Diary | 7d | 7d | Median Difference (%); Correlation Coefficient (S) |
| Little  (Nurse Questions)  (1999) | Interview  >51 to 99 food items / questions | Royal Society of Cambridge Database | Little  (1999) | Micronutrients (1); Food Groups | Adults & Elderly (18-80 yr)  *111 (53/58)* | Weighed food diary | 1d | 7d | Median Difference (%); Correlation Coefficient (S) |
| Margetts  (1989) | Self  >51 to 99 food items / questions | MCW4 | Margetts  (1989) | Energy;  Macronutrients (4); Micronutrients (4) | Adults (35-54 yr)  *433* | 24-hour recall | 1d | 1d | Correlation Coefficient (S); Cross Classification |
| Masson (Scottish Collaborative Group FFQ)  (2003) | Self  ≥100 food items / questions | UK National Nutrient Databank & MCW | Masson  (2003) | Energy;  Macronutrients (9); Micronutrients (15) | Adults (19-58 yr)  *81 (41/40)* | Weighed food diary | 1d | 4d | Relative Median Difference (%); Correlation Coefficient (S); Cross Classification; Weighed Cohen’s kappa |
|  |  |  | Heald  (2006) | Energy; Micronutrients (4) | Adults & Elderly (51–75 yr)  *203 (203/0)* | Biomarkers | 1d | 1d | Individual Medians; Correlation Coefficient (S); Cross Classification; Weighed Cohen’s kappa |
|  |  |  | Jia  (2008) | Energy;  Macronutrients (9); Micronutrients (15) | Elderly (64-80 yr)  *83 (42/41)* | Weighed food diary | 1d | 4d | Mean Difference; Correlation Coefficient (S); Cross Classification; Weighed Cohen’s kappa |
|  |  |  | Mohd-Shukri  (2013) | Energy; Macronutrients (10); Micronutrients (25) | Pregnant Women (21–45 yr)  *63 (0/63)* | Weighed Food Diary | 1d | 4d (Inc. 1 weekend d) | Individual Medians; Correlation Coefficient (P & S); Cross Classification; Weighted Cohen’s kappa. |
|  |  |  | Hollis  (2017) | Energy; Macronutrients (9); Micronutrients (16);  Food Group | Adults (18-65 yr)  *96 (40/56)* | Estimated Food Diary | 1d | 7d consecutive | Mean difference; Correlation Coefficient (S); Cross Classification; Limits of Agreement; Weighted Cohen’s kappa |
| McKeown (EPIC FFQ)  (2001) | Self  ≥100 food items / questions | MCW | McKeown  (2001) | Energy;  Macronutrients (7); Micronutrients (6);  Food Groups | Adults & Elderly (45-74 yr)  *146 (58/88) = Food Diary; 134 (57/77)=Biomarkers* | Weighed Food Diary; Estimated Food Diary & Biomarkers | 1d | 7d (Food Diary); 3 x 1d (Biomarkers) | Individual Means; Correlation Coefficient (P & S); Cross Classification |
|  |  |  | Day  (2001) | Micronutrients (2) | Adults & Elderly (45-74 yr)  *123* | Estimated Food Diary & Biomarkers | 1d | 7d (Food Diary); 6d over 12 months (Biomarkers) | Individual Means; Correlation Coefficient |
|  |  |  | Lietz  (2002) | Energy;  Macronutrients (6); Micronutrients (3) | Adolescents (11.8-13.2 yr)  *50 (32/18)* | Weighed Food Diary | 1d | 7d | Mean Difference; Correlation Coefficient (S); Cross Classification; Limits of Agreement |
| Mouratidou  (2006) | Self  >51 to 99 food items / questions | MCW5 | Mouratidou  (2006) | Energy;  Macronutrients(11); Micronutrients (24);  Food Groups | Pregnant Women (17-43 yr)  *123 (0/123)* | 24-Hour Recall | 1d | 2d | Individual Means; Correlation Coefficient (P); Cross Classification; Limits of Agreement |
| Nelson  (1988) | Interview  ≤50 food items / questions | MCW4 | Nelson  (1998) | Micronutrients | Elderly (65-90 yr)  *30 (0/30) = Food Diary; 28 (13/15) =Duplicate Diet* | Weighed Food Diary & Duplicate Diet | 2d (vs Food Diary); 1d (vs Duplicate Diet) | 7d (Food Diary); 5d (Duplicate Diet) | Individual Means; Correlation Coefficient; Cross Classification |
| O’Donnell  (1991) | Self  ≥100 food items / questions | DIET | O’Donnell  (1991) | Energy; Macronutrients (4); Micronutrients (14); | Adults (19-65 yr)  *52 (24/28)* | Weighed Food Diary & Biomarkers | 1d | 4 x 4d at 1 month intervals (Food Diary); 4d (Biomarkers) | Individual Means; Correlation Coefficient (P); Class Classification |
| Papadaki  (2007) | Self  ≤50 food items / questions | Not Reported | Papadaki  (2007) | Food Groups | Adults (25-55 yr)  *51 (0/51)* | Estimated Food Diary | 1d | 7d | Individual Means; Correlation Coefficient (P); Cross Classification; Limits of Agreement; Weighted Cohen’s kappa |
| Pufulete  (2002) | Self  >51 to 99 food items / questions | MCW5 | Pufulete  (2002) | Micronutrients (1) | Adults (22-65 yr)  *36 (16/20)* | Weighed Food Diary & Biomarkers | 2d | 7d | Individual Means; Correlation Coefficient; Cross Classification |
| Robinson  (2007) | By-Proxy  ≤50 food items / questions | MCW5 | Marriot  (2007) | Energy; Macronutrients (4); Micronutrients (18) | Infants (6 months)  *50 (25/25)* | Weighed Food Diary | 1d | 4d | Mean Difference (%); Correlation Coefficient (S); Limits of Agreement; |
| Robinson  (2007) | By-Proxy  ≤50 food items / questions | MCW5 | Marriot  (2007) | Energy; Macronutrients (4); Micronutrients (18) | Infants (12 months)  *50 (27/23)* | Weighed Food Diary | 1d | 4d | Mean Difference (%); Correlation Coefficient (S); Limits of Agreement; |
| Roddam  (2005) | Self  ≤50 food items / questions | MCW5 | Roddam  (2005) | Energy; Macronutrients (9); Micronutrients (12);  Food Groups | Adults (50-64 yr)  *202 (0/202)* | Weighed Food Diary & Estimated Food Diary | 2d | 7d | Median Difference (%); Correlation Coefficient (P); Cross Classification; Weighted Cohen’s kappa |
| Roe  (DINE)  (1994) | Interview  ≤50 food items / questions | MCW4 | Roe  (1994) | Energy  Macronutrients (4) | Adults (17–62 yr)  *206 (128/78)* | Estimated Food Diary | 1d | 4d | Correlation Coefficient (P); Cross Classification |
|  |  |  | Little  (1999) | Macronutrients (1); Micronutrients (1); Food Groups | Adults & Elderly (18-80 yr)  *111 (53/58)* | Weighed Food Diary | 1d | 7d | Median Difference (%) Correlation Coefficient (S); |
| Sofianou-Katsouilis  (2011) | By-Proxy  ≤50 food items / questions | Not Reported | Sofianou-Katsouilis  (2011) | Food Groups | Children (3-7 yr)  33 | 24-Hour Recall | 1d | 7d | Individual Means |
| Venter  (2006) | Not reported  ≤50 food items / questions | Not Reported | Venter  (2006) | Food Groups | Pregnant Women (20-44 yr)  *57 (0/57)* | Estimated Food Diary | 1d | 7d | Cross Classification; Cohen’s kappa |
| Yarnell  (1983) | Self  >51 to 99 food items / questions | MCW4 & MCW5 | Thompson  (1993) | Energy; Macronutrients (9); Micronutrients (6)  Food Group | Adults (40-59 yr)  *301 (122/ 179)*  Smokers only | Biomarkers | 1d | 10d | Mean Difference; Correlation Coefficient (S); Limits of Agreement |
|  |  |  | Bolton-Smith  (1991) | Micronutrients (5); | Adults (41-50 yr)  *196 (196/0)* | Biomarkers | 1d | Not Reported | Individual Means; Correlation Coefficient (P); Cross Classification; |

**Food Checklist**

| Bingham  (1994) | Self  ≥100 food items / questions | MCW4 | Bingham (pictures & no pictures) (1994) | Energy; Macronutrients (7); Micronutrients (6) | Adults (50-65 yr)  *160 (0/160)* | Weighed Food Diary | 7d | 4 x 4d | Individual Means; Correlation Coefficient (S); Cross Classification |
| --- | --- | --- | --- | --- | --- | --- | --- | --- | --- |
|  |  |  | Bingham ^[^(pictures & no pictures) (1997) | Micronutrients (3) | Adults (50-65 yr)  *160 (0/160)* | Biomarkers | 7d | 8d over 12 months | Correlation Coefficient (P & S) |
|  |  |  | Little  (no pictures) (1999) | Macronutrients (1); Micronutrients (1); Food Groups | Adults & Elderly (18-80 yr)  *111 (53/58)* | Weighed Food Diary | 7d | 7d | Median Difference (%); Correlation Coefficient (S) |
|  |  |  | Johansson  (no pictures) (2008) | Energy; Macronutrients (6); Micronutrients (6); Food Groups | Elderly (55-88 yr)  *80 (80/0)* | Weighed Food Diary | 4d | 4 x 4d over 12 months | Individual Means |
| Cade  (CADET)  (2006) | Self; By-Proxy  ≥100 food items / questions | DANTE | Cade  (2006) | Energy; Macronutrients (7); Micronutrients (5); Food Groups | Children (3-7 yr)  *180 (100/80)* | Semi-Weighed Food Diary | 1d | 1d | Mean Difference; Correlation Coefficient (S); Limits of Agreement |
|  |  |  | Christian  (2015) | Energy; Macronutrients (5); Micronutrients (3); Food Groups | Children (8-11 yr)  *67 (33/34)* | Weighed Food Diary | 1d | 1d | Mean Difference; Correlation Coefficient; Limits of Agreement |
| Johnson  (FIQ)  (1997) | **Self | Not reported | Johnson  (2001) | Food Groups | Adolescents (11-13 yr)  *93 (41/52)* | Estimated Food Diary | 1d | 3d | Correlation Coefficient (P) |
| Holmes  (2008) | Self; By-Proxy; Interview  ≥100 food items / questions | MCW5 | Holmes  (2008) | Energy; Macronutrients (4); Micronutrients (6);  Food Group | Children, Adolescents, Adults, Elderly (2-90 yr)  *76, 48, 206, 54*  Low SES | Weighed Food Diary | 4d | 4d | Mean Difference |

**Diet History**

| Black  (2000) | Interview | MCW4 | Black  (2000) | Energy; Macronutrients (2) | Adults (50-65 yr)  *64 (0/64)* | Weighed Food Diary; DLW; Biomarkers | 1d | 4 x 4d over 12 months (Food Diary); 8d over 12 months (Biomarkers); 14d (DLW) | Mean Difference; Correlation Coefficient (P); Limits of Agreement |
| --- | --- | --- | --- | --- | --- | --- | --- | --- | --- |
| Livingstone  (1992) | By Proxy; Interview | MCW4 | Livingstone  (1992) | Energy | Children & Adolescents (3-18 yr)  *78 (41/37)* | DLW | 1d | 10-14d | Mean Difference (%); Limits of Agreement |
| Jackson  (1990) | Interview | MCW4 | Jackson  (1990) | Macronutrients (2); Micronutrients (1) | Elderly (59-74 yr)  *80 (39/41)* | FFQ | 1d | 1d | Individual Means or Medians; Correlation Coefficient (P & S); Cross Classification; Weighted Cohen’s kappa |

** Studies that included multiple pass /days recall*

***Tool is web / smartphone based*

*MCW = McCance & Widdowson; DLW = Doubly Labelled Water; SES= Socio-economic status*

Table 4. Summary of validation results by reference method type, tool type and nutrient

| **Validation reference method / nutrient** | **Tool type** | **Number of validation study results^#^** | | | **Weighted mean differences*** | | | **Range of limits of agreement reported** | |
| --- | --- | --- | --- | --- | --- | --- | --- | --- | --- |
|  |  | **Infants, children and adolescents** | | **Adults and elderly** | **Infants, children and adolescents** | **Adults and elderly** | | **Infants, children and adolescents** | **Adults and elderly** |
| **Energy (kcal)** |  |  | |  |  |  | |  |  |
| Doubly labelled water | Food diary  Dietary Recall  FFQ / Food checklist | 3  3  0 | | 0  0  0 | -138  70  -- | --  --  - | | -1747 to 1045  -1102 to 879  -- | --  --  - |
| Food diary | Food diary  Dietary Recall  FFQ / Food checklist | 5  4  7 | | 6  9  19 | -18  254  247 | -46  -47  52 | | -1259 to 1261  -836 to 1628  -1497 to 1912 | -1223 to 1201  -1301 to 1706  -2036 to 2129 |
| 24-hour recall | Food diary  Dietary Recall  FFQ / Food checklist | 0  1  0 | | 1  1  2 | --  -55  -- | -52  3  366 | | --  -797 to 687  -- | -582 to 483  -1108 to 1113  -726 to 1480 |
| FFQ | Food diary  Dietary Recall  FFQ / Food checklist | 0  0  0 | | 0  0  1 | --  --  -- | --  --  671 | | --  --  -- | --  --  -523 to 1865 |
| **Protein (g)** |  |  | |  |  |  | |  |  |
| Biomarker* | Food diary  Dietary Recall  FFQ / Food checklist | 0  0  1 | | 1  0  1 | -  -  8.1 | 0.9  -  2.3 | | -  -  -3.5 to 19.7 | -5 to 6.8  -  -7 to 12 |
| Food diary | Food diary  Dietary Recall  FFQ / Food checklist | 5  4  7 | | 6  8  19 | 0.2  8.4  10.1 | -2.2  -0.9  6.0 | | -64 to 61  -40 to 61  -66 to 89 | -75 to 67  -67 to 79  -71 to 68 |
| 24-hour recall | Food diary  Dietary Recall  FFQ / Food checklist | 0  1  0 | | 1  1  2 | --  -2.0 | -4.0  -1.0  11.9 | | --  -45 to 41 | -34 to 26  -47 to 45  -39 to 70 |
| FFQ | Food diary  Dietary Recall  FFQ / Food checklist | 0  0  0 | | 0  0  1 | --  --  -- | --  --  -21.0 | | --  --  -- | --  --  -36 to 78 to 37 |
| **Carbohydrate(g)** |  |  | | |  | | |  | |
| Food diary | Food diary  Dietary Recall  FFQ / Food checklist | 5  4  7 | 6  8  19 | | -5.6  30.2  36.2 | -10.9  -8.7  18.5 | | -185 to 192  -132 to 229  -238 to 305 | -211 to 172  -161 to 196  -240 to 209 |
| 24-hour recall | Food diary  Dietary Recall  FFQ / Food checklist | 0  1  0 | 1  1  2 | | --  -11.0  -- | -2.0  -5.0  35.1 | | --  -152 to 130  -- | -98 to 94  -149 to 139  -112 to 177 |
| FFQ | Food diary  Dietary Recall  FFQ / Food checklist | 0  0  0 | 0  0  1 | | --  --  -- | --  --  -85.0 | | --  --  -- | --  --  -66 to 236 |
| **Total sugars (g)** |  |  | | |  | | |  | |
| Food diary | Food diary  Dietary Recall  FFQ / Food checklist | 0  0  2 | 1  2  14 | | --  --  38.7 | | 1.0  0.5  12.4 | --  --  -129 to 200 | -45 to 47  -74 to 86  -114 to 122 |
| 24-hour recall | Food diary  Dietary Recall  FFQ / Food checklist | 0  1  0 | 0  1  1 | | --  -14.0  -- | | --  -4.0  -6.0 | --  -121 to 92  -- | --  -92 to 83  -86 to 98 |
| FFQ | Food diary  Dietary Recall  FFQ / Food checklist | 0  0  0 | 0  0  1 | | --  --  -- | | --  --  -26.0 | --  --  -- | --  --  -42 to 94 to 42 |
| **Fat (g)** |  |  | | |  | | |  | |
| Food diary | Food diary  Dietary Recall  FFQ / Food checklist | 5  4  7 | 6  9  20 | | -0.03  11.8  8.6 | | 1.6  -0.5  -4.3 | -58 to 64  -50 to 88  -75 to 99 | -51 to 60  -71 to 87  -99 to 71 |
| 24-hour recall | Food diary  Dietary Recall  FFQ / Food checklist | 0  1  0 | 1  1  2 | | --  -3.0  -- | | -3.0  4.0  19.6 | --  -52 to 46  -- | -35 to 29  -62 to 69  -39 to 80 |
| FFQ | Food diary  Dietary Recall  FFQ / Food checklist | 0  0  0 | 0  0  1 | | --  --  -- | | --  --  -23.0 | --  --  -- | --  --  -32 to 78 to 31 |
| **Dietary fibre (g)** |  |  |  | |  | |  |  |  |
| Food diary | Food diary  Dietary Recall  FFQ / Food checklist | 0  0  3 | 2  3  7 | | --  --  2.6 | | -0.2  -0.1  2.5 | --  --  -19 to 23 | -8 to 7  -13 to 17  -13 to 19 |
| 24-hour recall | Food diary  Dietary Recall  FFQ / Food checklist | 0  1  0 | 0  1  2 | | --  -1.0  -- | | --  1.0  4.8 | --  -10 to 8  -- | --  -12 to 15  -6 to 19 |
| FFQ | Food diary  Dietary Recall  FFQ / Food checklist | 0  0  0 | 0  0  0 | | --  --  -- | | --  --  -- | --  --  -- | --  --  -- |
| **Retinol (µg)** |  |  |  | |  | |  |  |  |
| Biomarkers | Food diary  Dietary Recall  FFQ / Food checklist | 0  0  0 | 0  0  2 | | --  --  -- | | --  --  121 | --  --  -- | --  --  -979 to 1153 |
| Food diary | Food diary  Dietary Recall  FFQ / Food checklist | 0  0  0 | 2  2  8 | | --  --  -- | | 95.1  89.0  71.9 | --  --  -- | -2084 to 2226  -7360 to 7906  -2410 to 2450 |
| 24-hour recall | Food diary  Dietary Recall  FFQ / Food checklist | 0  0  0 | 0  0  1 | | --  --  -- | | --  --  92.4 | --  --  -- | --  --  341 to 526 |
| FFQ | Food diary  Dietary Recall  FFQ / Food checklist | 0  0  0 | 0  0  1 | | --  --  -- | | --  --  60.0 | --  --  -- | --  --  -425 to 545 |
| **Vitamin C (mg)** |  |  | | |  | | |  | |
| Biomarkers | Food diary  Dietary Recall  FFQ / Food checklist | 0  0  0 | 0  0  2 | | --  --  -- | | --  --  26.9 | --  --  -- | --  --  -32 to 80 |
| Food diary | Food diary  Dietary Recall  FFQ / Food checklist | 4  4  5 | 6  8  20 | | -2.5  16.5  16.5 | | -5.4  -1.0  54.9 | - 147 to 145  -108 to 154  -168 to 216 | -169 to 155  -159 to 197  -164 to 349 |
| 24-hour recall | Food diary  Dietary Recall  FFQ / Food checklist | 0  0  0 | 0  1  1 | | --  --  -- | | --  -7.0  -0.7 | --  --  -- | --  -202 to 188  -97 to 96 |
| FFQ | Food diary  Dietary Recall  FFQ / Food checklist | 0  0  0 | 0  0  1 | | --  --  -- | | --  --  57.4 | --  --  -- | --  --  -70 to 185 |
| **Calcium (mg)** |  |  | | |  | | |  | |
| Food diary | Food diary  Dietary Recall  FFQ / Food checklist | 4  4  7 | 6  8  21 | | 8.7  87.0  76.7 | | -48.3  -20.6  38.0 | -663 to 630  -565 to 744  -673 to 836 | -767 to 597  -822 to 873  -1003 to 1142 |
| 24-hour recall | Food diary  Dietary Recall  FFQ / Food checklist | 0  0  0 | 0  1  2 | | --  --  -- | | --  -8.8  111 | --  --  -- | --  -686 to 668  -646 to 769 |
| FFQ | Food diary  Dietary Recall  FFQ / Food checklist | 0  0  0 | 0  0  1 | | --  --  -- | | --  --  -324 | --  --  -- | --  --  -467 to 1115 to 467 |
| **Iron(mg)** |  |  | | |  | | |  | |
| Food diary | Food diary  Dietary Recall  FFQ / Food checklist | 4  4  5 | 6  8  20 | | -0.7  0.7  1.1 | | -0.7  -0.1  0.3 | -9.6 to 7.2  -6.6 to 9.4  -7.7 to 8.0 | -10.3 to 8.5  -11.9 to 13.3  -14 to 13.4 |
| 24-hour recall | Food diary  Dietary Recall  FFQ / Food checklist | 0  0  0 | 0  1  2 | | --  --  -- | | --  0.4  2.5 | --  --  -- | --  -9.1 to 9.9  -5.7 to 11.2 |
| FFQ | Food diary  Dietary Recall  FFQ / Food checklist | 0  0  0 | 0  0  1 | | --  --  -- | | --  --  6.2 | --  --  -- | --  --  -4 to 17 |
| **Folate (µg)** |  |  | | |  | | |  | |
| Food diary | Food diary  Dietary Recall  FFQ / Food checklist | 4  4  5 | 5  6  15 | | -10.7  11.3  31.4 | | -17.2  -6.5  70.9 | -309 to 259  -257 to 263  -268 to 300 | -497 to 451  -307 to 417  -244 to 336 |
| 24-hour recall | Food diary  Dietary Recall  FFQ / Food checklist | 0  0  0 | 0  1  2 | | --  --  -- | | --  24.5  48.4 | --  --  -- | --  -214 to 263  -106 to 205 |
| FFQ | Food diary  Dietary Recall  FFQ / Food checklist | 0  0  0 | 0  0  1 | | --  --  -- | | --  --  -125 | --  --  -- | --  --  -106 to 356 |
| **Sodium (mg)** |  |  | | |  | | |  | |
| Biomarker | Food diary  Dietary Recall  FFQ / Food checklist | 0  0  0 | 1  0  1 | | --  --  -- | | --572  --  -575 | --  --  -- | -3103 to 1960  --  -3875 to 2725 |
| Food diary | Food diary  Dietary Recall  FFQ / Food checklist | 0  0  2 | 0  0  6 | | --  --  571 | | --  --  -190 | --  --  -2879 to 3715 | --  --  -3956 to 2620 |
| 24-hour recall | Food diary  Dietary Recall  FFQ / Food checklist | 0  1  0 | 0  0  1 | | --  -20.0  -- | | --  --  106 | --  -2900 to 2900  -- | --  --  -2048 to 2260 |
| FFQ | Food diary  Dietary Recall  FFQ / Food checklist | 0  0  0 | 0  0  1 | | --  --  -- | | --  --  -155 | --  --  -- | --  --  -1615 to 1926 |
| **Zinc (mg)** |  |  |  | |  | |  |  |  |
| Food diary | Food diary  Dietary Recall  FFQ / Food checklist | 0  0  0 | 0  0  4 | | --  --  -- | | --  --  1.7 | --  --  -- | --  --  -10 to 9 |
| 24-hour recall | Food diary  Dietary Recall  FFQ / Food checklist | 0  0  0 | 0  0  1 | | --  --  -- | | --  --  1.6 | --  --  -- | --  --  -4 to 7 |
| FFQ | Food diary  Dietary Recall  FFQ / Food checklist | 0  0  0 | 0  0  0 | | --  --  -- | | --  --  -- | --  --  -- | --  --  -- |

**Nitrogen values, not protein values*

*^#^ Results for different age groups and genders within the two main age groups were taken into account separately.*

** Weighted mean differences between the intakes = test tool mean intake minus reference method mean intake; these were weighted using the number of individuals taking part in each validation studies to calculated the overall mean difference for each validation and tool type combination .*

Supplementary material

Supplementary table 1. Definition of the main types of dietary assessment tools

| **Name of tool** | **Description** | **Time duration** |
| --- | --- | --- |
| Weighed food diary | Each food and beverage item consumed is described, weighed and recorded using recognised units of measurement (e.g. grams, fluid ounces) with leftovers also being recorded. | Usually between 3-7 days but can be longer |
| Semi-weighed food diary | Some food and beverage items are weighed using household units of measurement (e.g. cups, spoons) and / or photographs. Estimates are converted into weights for analysis | Usually between 3-7 days but can be longer |
| Estimated food diary | Each food and beverage is estimated rather than weighed by using standard household units of measurement (e.g. cups, spoons) and / or photographs or food models. Estimates are subsequently converted into weights for analysis | Usually between 3-7 days but can be longer |
| 24-hour recall | Participants are interviewed by a trained practitioner where they are asked to recall the food and beverage items, along with portion size and quantity, consumed over the previous 24-hours. | 24-hours |
| 48-hour recall | Participants are interviewed by a trained practitioner where they are asked to recall the food and beverage items, along with portion size and quantity, consumed over the previous 48 hours | 48-hours |
| Multiple pass / days recall | A 4-stage process where a trained practitioner asks participants to recall food and beverage items, along with portion size and quantity. Stage 1 requires obtaining a list of consumed foods. Stage 2 involves information (time, place,) on consumption. Stage 3 involves recalling any foods that may have been forgotten. Stage 4 obtains details on portion sizes and an overall review of answers | Can range from 1 – 5 days |
| Food frequency questionnaire (FFQ) | A questionnaire that contains a list of foods and frequency of consumption usually via a 'tick box' option. | Can range from less than a week up to 1 year |
| Semi-quantitative FFQ | Similar to standard food frequency questionnaire with the addition of portion sizes being recorded in addition to consumption frequency | Can range from less than a week up to 1 year |
| Food checklist | Where participants mark the foods consumed from a list along with serving size and time of consumption. Examples include the paper based CADET tool ^[77]^_]_ assessing primary school children’s diet and web-based Food Intake Questionnaire investigating adolescent dietary habits ^[31]^. | Usually across 3-5 days but can be used for one day |
| Diet history | Consists of questions about participant eating habits along types of foods / beverages consumed and frequency | Can range from less than a week up to 1 year |

Supplementary table 2 General characteristics of the reviews that reported dietary assessment tools

| **First Author and year** | **Life stage** | **Dietary Exposure Studied** | **Dietary Tool Reviewed** | **Month / year range of published articles included** | **No of articles included in review** | **No of potentially eligible validated dietary assessment tools** | **Systematic (S) review** |
| --- | --- | --- | --- | --- | --- | --- | --- |
| Arens-Volland, A.G.  (2015) | All | All assessed* | Computer-based | <04/2014 | 32 | 1 |  |
| Bach, A.  (2006) | All | Mediterranean Diet | Dietary Indexes | Unknown | 27 | 0 |  |
| Bell, L. K.  (2013) | Infants & Children | All assessed* | Brief Tools | ≤04/2013 | 15 | 1 | Y |
| Bertin, R.L  (2006) | Pregnant women | All assessed* | All | 1994-09/2004 | 14 | 0 |  |
| Borges, C. A.  (2015) | All | All assessed* | Dietary patterns | 1980-2012 | 189 | 2 |  |
| Bryant, M.  (2014) | Infant, Children & Adolescents | All assessed* | All | 1960 – 10/2011 | 44 | 1 | Y |
| Burrows, T.  (2012) | Children & Adolescents (<20 years old) | All assessed* | All | 1985 - 08/2010 | 31 | 0 | Y |
| Burrows, T.  (2010) | Infant, Children & Adolescents | Energy | All | 1973 - 01/2009 | 15 | 6 | Y |
| Cade, J. E.  (2004) | All | All assessed* | FFQs | 1980 - 09/1999 | 219 | 24 |  |
| Calfas, K. J.  (2000) | All | All assessed* | Brief Tools | ≤2000 | 14 | 1 |  |
| Carvalho, K.  (2014) | All | All assessed* | Dietary Indexes | ≤06/2014 | 20 | 0 |  |
| Contento, I. R.  (2002) | All | All assessed* | All | 1980 - 1999 | 156 | 0 |  |
| De Lauzon-Guillain, B  (2012) | Infant & Children (0-5years) | All assessed* | All | ≤03/2010 | 19 | 1 | Y |
| England, C. Y.  (2015) | All | All assessed* | Brief Questionnaires | ≤06/2013 | 35 | 1 | Y |
| Falcão-Gomes, R. C.  (2006) | Infants & Children (≤7 years old) | All assessed* | All | 1997 - 07/2005 | 33 | 0 |  |
| Falomir, Z.  (2012) | All | All | Web-based & Computerized -FFQ/24hr Recall | 1980 - 12/2011 | 40 | 4 |  |
| Frainer, D.E.  (2008) | Children & Adolescents (6 – 18 years) | Energy | All | 1996 - 04/2006 | 10 | 0 | Y |
| Gavreila, A  (2014) | All | All assessed* | Web-based - 24hr Recall & Food Record | 1995 - 05/2013 | 58 | 6 |  |
| Henriquez-Sanchez, P.  (2009) | All | Vitamins | All | ≤03/2008 | 124 | 15 | Y |
| Illner, A. K.  (2012) | All | All assessed* | Innovative technologies | 01/1995 - 09/2011 | 29 | 2 |  |
| Kolodziejczyk, J. K.  (2012) | Children & Adolescents (6-18 years) | Foods/Food groups | FFQs | 01/2001 - 12/2010 | 21 | 0 | Y |
| Kourlaba, G.  (2009) | All | All assessed* | Dietary Quality Indices | ≤06/2008 | 38 | 0 |  |
| Kim, D. J.  (2003) | Adults | Fruit & Vegetables | Brief survey instruments | 1980 - 2003 | 10 | 1 |  |
| Lambert, J.  (2004) | Children & Adolescents | All assessed* | All | ≤12/2001 | 87 | 1 |  |
| Lee, H.  (2016) | All | All assessed* | FFQ | 1983 – 05/2014 | 277 | 9 | Y |
| Lombard, M. J.  (2015) | All | All | All | 01/2009 – 12/2014 | 60 | 0 |  |
| Long, J. D.  (20101) | All | Include Fruit and Vegetable | Technology-based | 1998 - 2008 | 15 | 0 |  |
| McPherson, R. S.  (2000) | Children | All assessed* | All | 01/1970 – 04/1999 | 47 | 0 |  |
| Magarey, A.  (2015) | Adults | Calcium | All | 1948 - 02/2013 | 36 | 4 | Y |
| Marshall, S.  (2014) | Children & Adolescents | All assessed | Diet Quality Indices | 1980 – 10/2013 | 119 | 0 | Y |
| Molag, M. L.  (2007) | All | All assessed* | FFQs | 1980 – 12/2006 | 40 | 10 |  |
| Moran, V. H.  (2007) | Pregnant Adolescents | All assessed* | All | 1980 - 2006 | 9 | 0 | Y |
| Mouratidou, T.  (2012) | Children (3-9 years) | All assessed* | All | 1990 - 2010 | 25 | 1 |  |
| Ngo, J.  (2009) | All | All assessed* | Computerized methods | 01/1995 – 02/2008 | 16 | 0 |  |
| Olukotun, O.  (2015) | Infant & Children (<11 years) | All assessed* | All | 1994 - 2014 | 9 | 3 | Y |
| Ortiz-Andrellucchi, A.  (2009) | Infants, Children & Adolescents | Micronutrient | All | <04/2008 | 32 | 6 | Y |
| Ortiz-Andrellucchi, A.  (2009) | Elderly | Micronutrient | All | <04/2008 | 33 | 2 | Y |
| Ortiz-Andrellucchi, A.  (2009) | Pregnant Women | Micronutrient | All | Unknown | 17 | 3 | Y |
| Overby, N. C.  ^(2009)^ | All | n-3 fatty acid | All | ≤03/2008 | 14 | 0 | Y |
| Palmer, M. A.  (2012) | Adults | Dietary iron | FFQs | ≤04/2010 | 9 | 1 |  |
| Park, J. Y.  (2013) | All | Folate | All | ≤09/2011 | 17 | 1 |  |
| Pedraza, D. F.  (2015) | All | All assessed* | FFQs | <2013 | 41 | 0 |  |
| Probst, Y. C.  (2005) | All | All assessed* | Computerized programs | 1996 - 2003 | 33 | 6 |  |
| Rankin, D.  (2010) | Adolescents | All assessed* | All | 1990 - 2009 | 21 | 1 |  |
| Riordan, F  (2016) | All | Sugar-sweetened beverages | All | 1990 – 06/2014 | 69 | 2 | Y |
| Riordan, F  (2016) | All | Fruit & vegetables | All | 1990 – 07/2014 | 108 | 3 | Y |
| Rodrigues, A. G. M.  (2011) | All | All assessed* | Tools that use food images | <2011 | 21 | 0 |  |
| Romas-Vinas, B.  (2009) | All | Dietary Patterns | All | ≤04/2008 | 19 | 0 |  |
| Roman-Vinas, B.  (2010) | Infants, Children & Adolescents | Micronutrient | FFQ | <06/2008 | 19 | 3 | Y |
| Sasaki, S.  (2003) | Japanese | All assessed* | FFQs | 1990 - 05/2003 | 24 | 0 |  |
| Serdula, M. K.  (2001) | Infant & Children (Preschool) | All assessed* | All | 01/1976 - 08/2000 | 25 | 3 |  |
| Serra-Majem, L.  (2009) | All | Micronutrient (iron, calcium, selenium, zinc & iodine) | All | <03/2008 | 109 | 13 | Y |
| Serra-Majem, L.  (2012) | All | omega-3 fatty acid | All | ≤05/2011 | 19 | 0 | Y |
| Sharp, D. B.  (2014) | All | All assessed* | Mobile phones | 01/2001 -08/2013 | 15 | 1 |  |
| Silva, T. D. A.  (2012) | All | All assessed | FFQ | 1998 - 2010 | 22 | 0 | Y |
| Sutton, E.  (2008) | Infants | Sodium | All | Unknown | 5 | 0 |  |
| Tabacchi, G.  (2014) | Adolescents | All assessed* | All | 2001 - 2011 | 20 | 2 | Y |
| Tabacchi, G.  (2016) | Adolescents (13-17 years) | All assessed | FFQs | 2001 - 2012 | 16 | 1 | Y |
| Trabulsi, J.  (2001) | All | Energy | All | Unknown | 19 | 3 |  |
| Timmins, K.  (2014) | All | All assessed* | New technologies | 2000 - 05/2014 | 148 | 4 | Y |
| Vezina-Im, L. A.  (2014) | Pregnant Women | All assessed* | All | ≤31/01/2014 | 56 | 3 | Y |
| Vucic, V.  (2009) | Low-income Population | All assessed* | All | 1990 – 03/2008 | 7 | 3 |  |
| Wakai, K.  (2009) | Japanese | All assessed* | FFQ | 1980 – 06/2008 | 21 | 0 |  |
| Walker, J. L.  (2011) | Infant & Children (0-5 years) - with cerebral palsy | Energy | All | 1966 - 02/2011 | 4 | 0 |  |
| Wirt, A.  (2009) | All | All assessed* | Diet Quality tools | 2004 - 2007 | 36 | 0 |  |
| Wojtusiak, J.  (2011) | African | All assessed* | All | Unknown | 8 | 0 |  |
| Yang, W.  (2014) | Infant, Children & adolescents | All assessed* | All | ≤09/2011 | 16 | 0 | Y |
| Yaroch, A. L.  (2000) | All | Dietary Fat | Dietary Index Questionnaires | Unknown | 16 | 0 |  |

**All assessed refers to all main macronutrients (protein, carbohydrate, fat, dietary fibre) and some key micronutrients*

# Appendix 1.

1     exp diet/

2     Nutritional status.mp.

3     diet* adj2 intake*.mp

4    diet* adj2 qualit*.mp.

5     food adj2 intake*.mp.

6     nutri* adj2 intake*.mp.

7     diet* adj2 habit*.mp.

8 food adj2 habit.mp.

9     diet* pattern* or meal pattern*.mp.

10   food group*.mp.

11   nutrient*.mp.

12   macro-nutrient* or macronutrient.mp.

13   micro-nutrient or micronutrient.mp.

14   energy intake*.mp.

15   1 or 2 or 3 or 4 or 5 or 6 or 7 or 8 or 9 or 10 or 11 or 12 or 13 or 14

16   diet* adj2 (method* or tool* or survey* or record* or assess*).mp.

17   diet* adj2 (recall* or questionnaire* or histor* or instrument*).mp.

18   nutrition* adj2 (survey* or assess* or instrument*).mp. (27252)

19   food adj2 (questionnaire* or record* or recall* or diar* or checklist* or screener*).mp

20   24* adj2 recall.mp.

21   multiple pass.mp

22   FFQ*.mp

23   diet* adj2 (measure* or analys*).mp

24   nutri* adj2 measur*.mp

25   16 or 17 or 18 or 19 or 20 or 21 or 22 or 23 or 24

26   valid*.mp.

27   reliab*.mp.

28   reproduc*.mp.

29   calibrat*.mp.

30 repeatab*.mp

31 feasib*.mp

32 evaluat*.mp

33   26 or 27 or 28 or 29 or 30 or 31

34   review*.mp

35   meta-analy*.mp.

36   search*.mp.

37   systematic* adj2 (approach or analys*).mp.

38   33 or 34 or 35 or 36

39     15 and 25 and 32 and 37
